# Effects of Coenzyme Q10 Supplementation on Muscle Health and Physical Capacity in Middle-Aged and Older Adults: A Systematic Review and Meta-Analysis of Randomized Controlled Trials

**DOI:** 10.3390/antiox15101219

**Published:** 2026-09-22

**Authors:** Xundian Liu, Jinxuan Bao, Zhuoxuan Yu, Wende Zheng, Yaxuan Huang, Yifan Zhang, Xiuying Jiang

**Affiliations:** 1 Sports Coaching College, Beijing Sport University, Beijing 100084, China; 2023010561@bsu.edu.cn (X.L.);; 2 School of Strength and Conditioning Training, Beijing Sport University, Beijing 100084, China; baojinxuan@bsu.edu.cn; 3 College of Education, Beijing Sport University, Beijing 100084, China; 4 School of Sports Training, Tianjin University of Sport, Tianjin 301617, China; 5 Sports Department, Tianjin University, Tianjin 300354, China; 6 Division of Sport Anatomy, School of Sport Science, Beijing Sport University, Beijing 100084, China; 7 Laboratory of Sports Stress and Adaptation of General Administration of Sport, Beijing 100084, China

**Keywords:** coenzyme Q10, ageing, aerobic capacity, muscle strength, physical function, randomized controlled trial, systematic review, meta-analysis

## Abstract

Age-related declines in physical capacity may partly reflect impaired mitochondrial bioenergetics and oxidative stress. This systematic review and meta-analysis evaluated the effects of oral coenzyme Q10 (CoQ10) supplementation on muscle health and physical capacity in adult populations with a study-level mean or median age ≥ 40 years. MEDLINE, Embase, Web of Science, Scopus, and CENTRAL were searched through July 2026 for randomized controlled trials lasting ≥2 weeks. Directly usable evidence formed primary random-effects models, whereas assumption-dependent estimates were included only in sensitivity analyses. Effect measures were expressed as mean differences (MDs) or Hedges’ g, as appropriate. Thirty-three trials involving 3317 randomized participants were included, and 21 contributed to meta-analysis. The relative VO_2_peak/VO_2_max estimate was small and imprecise (5 studies, n = 172; MD 0.73 mL·kg^−1^·min^−1^, 95% CI −0.07 to 1.54; I^2^ = 0%; low certainty) and remained compatible with no benefit. Primary estimates were also uncertain for 6-min walking distances, handgrip strength, lower-limb strength, chair-rise performance, exercise duration, and muscle/lean-tissue quantity. Sensitivity analyses did not materially alter the overall interpretation. Evidence certainty was low or very low for all key outcomes. Current evidence does not establish clinically meaningful benefits of CoQ10 supplementation for aerobic capacity, muscle health, or physical function.

## 1. Introduction

Population ageing has increased the number of adults living with progressive losses of skeletal muscle strength, muscle quantity and quality, and cardiorespiratory fitness. These changes may begin during middle age and become increasingly pronounced in later life, particularly in the presence of chronic disease, physical inactivity, inadequate nutrition, or long-term pharmacological treatment. The revised European consensus on sarcopenia identifies low muscle strength as the primary characteristic of sarcopenia, with reduced muscle quantity or quality used to confirm the diagnosis and impaired physical performance indicating greater severity [1]. This diagnostic framework is relevant to the present review because it distinguishes muscle strength, muscle quantity or quality, and physical performance as related but non-interchangeable domains that map directly onto the outcomes evaluated here; it was used to organize the review outcomes rather than to imply that all included participants had clinically diagnosed sarcopenia. Longitudinal evidence further suggests that muscle strength declines more rapidly than lean mass, indicating that age-related functional deterioration cannot be explained by muscle loss alone [2]. Aerobic capacity also decreases throughout adulthood, with an accelerated decline in peak oxygen uptake during later decades of life [3]. Together, these functional losses provide the clinical rationale for evaluating CoQ10, a mitochondrial electron carrier and antioxidant, as a potential intervention for preserving mobility, exercise tolerance, and independent living during middle and older age.

Age-related deterioration in physical function has been associated with alterations in skeletal-muscle mitochondrial energetics, oxidative capacity, and quality control, although these relationships are also influenced by habitual physical activity and cardiometabolic health [4]. Coenzyme Q10 (CoQ10), present mainly as ubiquinone and its reduced form ubiquinol, is an endogenously synthesized lipid-soluble electron carrier and redox-active molecule involved in mitochondrial energy production and cellular antioxidant defence [5]. Altered CoQ10 status has been reported in some age-related and chronic clinical conditions [6].

Despite this mechanistic rationale, the clinical effects of CoQ10 supplementation on muscle and physical outcomes remain uncertain. Randomized trials have investigated CoQ10 across heterogeneous populations, including healthy adults, older individuals, physically trained participants, statin users, and patients with cardiovascular, metabolic, or neuromuscular conditions. Reported outcomes have included maximal or peak oxygen uptake, walking capacity, exercise duration, maximal workload, time to exhaustion, handgrip strength, lower-limb strength, chair-rise performance, mobility, fatigue, and indices of muscle quantity. However, the direction and magnitude of the reported effects have not been consistent. Potential sources of variation include differences in baseline CoQ10 status, age and health status, supplementation dose, ubiquinone or ubiquinol formulation, intervention duration, concomitant exercise training, and the sensitivity of the physical-performance tests employed. Recent evidence indicates that supplementation reliably increases circulating CoQ10 concentrations, whereas improvements in exercise performance are generally smaller, less consistent, and dependent on the population and outcome examined [7,8]. Evidence concerning exercise-induced oxidative stress and muscle damage also does not necessarily translate into measurable improvements in aerobic capacity, muscle strength, or habitual physical function [7]. Consequently, biochemical responsiveness should not be assumed to represent clinically meaningful functional benefit.

Several previous systematic reviews have examined nutritional supplementation and physical function in older people or the effects of CoQ10 on exercise-related outcomes. A broad review of nutritional interventions in older adults evaluated multiple nutrients rather than isolating the effects of CoQ10 [9]. More recent CoQ10 meta-analyses have predominantly combined adults across a wide age range, focused on healthy or physically active populations, or emphasized exercise-induced oxidative stress, biomarkers of muscle damage, and short-term exercise performance [7,8]. Although these studies provide important evidence, they do not specifically resolve whether CoQ10 supplementation improves clinically relevant domains of muscle health and physical capacity in middle-aged and older adults across both healthy and clinical populations. In addition, previous syntheses have not always clearly distinguished directly reported results from estimates that required additional statistical assumptions. This limits assessment of whether pooled conclusions are robust to data-recovery decisions and provides a rationale for separating primary evidence from assumption-dependent sensitivity evidence in the present review.

Therefore, the present systematic review and meta-analysis aimed to evaluate the effects of oral CoQ10 supplementation on muscle health and physical capacity in adult populations with a study-level mean or median age of ≥40 years, or in separately extractable subgroups meeting this threshold. Only randomized controlled trials comparing CoQ10 with placebo, no supplementation, or an otherwise equivalent control condition were considered. Outcomes of interest encompassed aerobic capacity, walking and functional capacity, exercise performance, muscle strength and function, muscle quantity, and validated clinician-rated disease-specific motor function. Evidence was organized into clinically coherent outcome domains, with directly supported estimates used in primary analyses and assumption-dependent estimates reserved for sensitivity analyses. Disease-specific motor scales were summarized within their clinical context and were not combined across neurological disorders. By examining effect magnitude, precision, heterogeneity, risk of bias, robustness, and certainty, this review sought to clarify the potential role of CoQ10 supplementation in preserving muscle and physical function during middle and older age and to identify priorities for future randomized trials.

## 2. Methods

### 2.1. Protocol, Registration, and Reporting

This systematic review and meta-analysis were reported according to PRISMA 2020 [10] and registered in PROSPERO (CRD420261469564). An internal statistical analysis plan (SAP) was finalized before completion of the final outcome-level extraction, final effect-size calculations, and pooled modelling. The SAP prespecified the A/B/C evidence-routing framework, outcome-selection and data-recovery rules, and planned sensitivity and subgroup analyses. Routing was based on quantitative recoverability, required assumptions, and clinical comparability, independently of effect magnitude, direction, statistical significance, RoB 2 judgment, or GRADE certainty. Post-SAP changes were documented in an amendment log, and a completed PRISMA 2020 checklist is provided separately. Consistent with contemporary transparency principles for systematic-review reporting [11] and established approaches to the statistical recovery of incomplete summary data [12,13], this operational routing framework makes the influence of data-recovery decisions auditable: directly supported estimates determine the primary analyses, whereas estimates requiring statistical reconstruction or unverifiable assumptions are confined to prespecified sensitivity analyses. The A/B/C labels are review-specific operational categories rather than a formally endorsed classification system.

### 2.2. Eligibility Criteria

Eligible populations were adults from studies with a reported study-level mean or median age ≥ 40 years, or separately extractable subgroups meeting this threshold; both nonclinical and chronic-disease populations were eligible. Interventions comprised oral CoQ10 (including ubiquinone, ubiquinol, ubidecarenone, and clearly identified enhanced-bioavailability formulations) at any dose for ≥2 weeks. Balanced co-interventions were permitted, and multi-ingredient, factorial, or multi-arm trials were eligible only when the independent CoQ10 effect could be isolated. Comparators included placebo, no supplementation, usual care, or an otherwise equivalent background intervention.

Eligible outcomes were objective measures of aerobic capacity, walking or functional capacity, exercise performance, muscle strength or function, muscle/lean-tissue quantity, or validated clinician-rated disease-specific motor or functional outcomes. Biochemical markers, subjective fatigue, quality of life, pain, and other patient-reported outcomes alone were insufficient for eligibility. Randomized parallel-group, crossover, and factorial designs were eligible when random allocation was verifiable; crossover trials additionally required randomized treatment sequence. Non-randomized and quasi-randomized studies, single-arm or observational studies, acute non-supplementation experiments, protocols, reviews, case reports, and animal or in vitro studies were excluded.

Systematic-review eligibility was assessed separately from quantitative usability: otherwise eligible trials were retained when numerical results required conversion, imputation, graphical recovery, crossover assumptions, or narrative synthesis. Multiple reports from one randomized trial were linked and treated as a single study. Detailed PICOS criteria and boundaries are provided in Supplementary Methods S1.

### 2.3. Information Sources and Search Strategy

MEDLINE via PubMed (20 July 2026), Embase (21 July 2026), Web of Science Core Collection (21 July 2026), Scopus (21 July 2026), and CENTRAL (21 July 2026) were searched from inception without language or publication-year restrictions, following PRISMA-S reporting principles [11]. PubMed used a broad CoQ10-plus-randomized-trial strategy without mandatory age or outcome terms; Embase added age and outcome blocks, Web of Science and Scopus added outcome terms, and CENTRAL combined CoQ10 and outcome concepts within the Trials collection. The final executed strategies were retained as run rather than reconstructed retrospectively. After study selection was complete, a post hoc known-item recovery audit used the 33 final included reports as the benchmark set. Reports were matched against the original, unmodified database exports by DOI when available and by normalized title otherwise; recovery was calculated separately for each database and for their union. Because the benchmark comprised studies identified within the completed review, this audit assessed recovery of known eligible reports and was not interpreted as proof of absolute sensitivity for unidentified or unindexed studies. Reference lists of eligible trials and relevant reviews were also checked. Exact executed strategies, limits, search dates, and pre-deduplication yields are reproduced in Supplementary Methods S1.

### 2.4. Study Selection

Search records were imported into EndNote and deduplicated using bibliographic identifiers and manual verification. Two reviewers independently screened titles/abstracts and then full texts against the prespecified criteria. At title/abstract screening, records were excluded only when both reviewers agreed that they clearly represented a non-randomized design, an ineligible age population without a separately extractable subgroup, an intervention from which the independent CoQ10 effect could not be isolated, an intervention lasting less than 2 weeks, or a study without an eligible objective muscle, physical-performance, physiological, or disease-specific motor outcome. Any uncertainty or a potentially eligible judgment by either reviewer triggered full-text assessment. One primary reason for exclusion was recorded for each excluded full text. Screening disagreements were resolved by consensus or, when necessary, third-reviewer adjudication. Multiple reports from the same trial were linked using study and intervention characteristics to prevent double counting. No records were excluded solely through automated screening tools.

### 2.5. Data Extraction and Outcome Hierarchy

Data were extracted with a standardized, piloted form by Xundian Liu and independently verified against the source reports by Jingxuan Bao. The two reviewers jointly rechecked the original report and resolved discrepancies through discussion; if a discrepancy remained unresolved, Xiuying Jiang served as the third reviewer for adjudication. Extracted information covered study and participant characteristics, intervention and comparator details, eligible outcomes, numerical data required for effect-size calculation, and attrition. Main results tables were preferred, followed by Supplementary Materials, companion reports, registries, and figures. Intention-to-treat and direct between-group estimates were preferred when available. Prespecified adjusted estimates were used when appropriate; otherwise, change scores with usable variance or post-intervention values were selected. Extracted and derived values were checked against sample sizes, confidence intervals, test statistics, and direction of benefit.

Outcomes were organized into clinically coherent domains: relative VO_2_peak/VO_2_max, walking and functional capacity, exercise duration and maximum workload, chair-rise performance, handgrip and lower-limb strength, muscle/lean-tissue quantity, and validated disease-specific motor or functional outcomes. Disease-specific scales were interpreted within their disorder and instrument context and were not pooled across neurological disorders or combined with instrumented physical-performance outcomes. Pooling therefore depended on outcome-level construct comparability rather than diagnosis alone.

Only one effect estimate from each independent study comparison entered a given meta-analysis. When multiple estimates represented the same domain, selection followed a concise hierarchy: the trial-designated primary outcome was preferred, followed by the most direct validated and cross-study-comparable measure based on the largest eligible analysis sample. Bilateral strength values were preferred or combined where possible. The end of the randomized intervention was the primary follow-up; when several assessments occurred during active treatment, the longest prespecified time point before discontinuation or crossover was used. Results requiring additional assumptions or recovery were routed to Class B sensitivity or standalone analysis rather than entering primary models. Class A primary inputs and the full operational selection rules are reported in Supplementary Table S2 and the statistical analysis plan.

### 2.6. Evidence Routing: Core, Sensitivity, and Narrative Synthesis

Each eligible outcome result was assigned an outcome-specific evidence class and analytic route according to the prespecified statistical analysis plan. Class A comprised directly reported or deterministically recoverable between-group estimates with usable variance and no unverifiable assumptions; clinically comparable Class A results formed the primary models, while non-exchangeable results were reported as A-standalone. Class B comprised estimates requiring a prespecified statistical assumption or recovery procedure, including median-to-mean conversion, assumed within-participant correlation, crossover reconstruction, or verified digitization; compatible Class B estimates entered A + B sensitivity models, while others were reported as B-standalone. Class B estimates were considered acceptable for sensitivity analysis because the recovery procedures were prespecified, transparent, based on conventional statistical relationships, and examined across plausible alternative assumptions. They were not allowed to determine the primary conclusions or formal GRADE ratings. Class C comprised eligible results for which no defensible quantitative estimate or variance could be recovered and was synthesized narratively.

Routing was independent of observed effect magnitude, direction, *p*-value, RoB 2, and GRADE. Evidence class therefore described quantitative recoverability rather than internal validity or certainty. At study level, each trial was counted once according to its highest class (A > B > C), whereas Supplementary Tables S2 and S3 provide the authoritative outcome-level routes.

### 2.7. Risk-of-Bias Assessment

Risk of bias was assessed with Cochrane RoB 2 [14] for the effect of assignment to intervention. Parallel-group trials were evaluated with the standard RoB 2 tool for individually randomized parallel-group trials (version 22 August 2019). Randomized crossover trials were evaluated with the dedicated RoB 2 variant for randomized crossover trials (version 18 March 2021), which adds Domain S for risk of bias arising from period and carryover effects. Domain S considered whether participants were allocated to treatment sequences in equal or nearly equal proportions, whether period effects were accounted for when required, and whether sufficient time elapsed for carryover to disappear before subsequent outcome measurement. Period and carryover concerns were therefore adjudicated in Domain S rather than being reassigned to Domain 2. Two reviewers assessed judgments independently and resolved disagreements by discussion or third-reviewer adjudication. Domain and overall judgments were categorized as low risk, some concerns, or high risk and were not converted to numerical scores or used for automatic exclusion. Because outcome-specific meta-analyses generally contained few studies, separate high-risk-study exclusion models were not considered sufficiently stable; risk of bias instead informed GRADE and interpretation, with individual-study influence examined through leave-one-out analyses when at least three studies were available.

### 2.8. Effect Measures and Statistical Synthesis

Continuous outcomes measured on a common scale were summarized as mean differences (MDs) with 95% confidence intervals: mL·kg^−1^·min^−1^ for relative VO_2_peak/VO_2_max, metres for 6-min walking distance, watts for maximum workload, kilograms for handgrip strength, and newton-metres for lower-limb strength. Hedges’ g was used for common constructs assessed with non-exchangeable scales or protocols, specifically exercise duration, chair-rise performance, and muscle/lean-tissue quantity. Disease-specific scales remained on their original scale and were combined only within the same disease, instrument, and comparable follow-up context. All effects were oriented so that positive values favoured CoQ10; estimates for outcomes in which lower values were better were sign-reversed accordingly.

Clinical poolability was determined before statistical synthesis from the outcome construct, measurement scale and protocol, assessment context, and interpretability of a common effect. Diagnosis alone did not preclude pooling when studies assessed the same underlying objective construct using sufficiently comparable measurement procedures and interpretable effect scales. Accordingly, relative VO_2_peak/VO_2_max, 6-min walking distance, handgrip strength, and comparable lower-limb strength measures could be pooled across eligible clinical and nonclinical populations when these criteria were met. In contrast, outcomes whose clinical meaning depended on a specific disorder or instrument, such as UPDRS, UHDRS, UMSARS, and PSP Rating Scale scores, were retained as disease-specific standalone evidence and were not pooled across disorders. Meta-analysis was performed only when at least two independent studies were sufficiently comparable.

Random-effects models used restricted maximum likelihood estimation with Hartung–Knapp inference [15]. Heterogeneity was described using Cochran’s Q, τ^2^, and I^2^ [16], considering study-specific effects and clinical or methodological differences rather than fixed I^2^ thresholds. Prediction intervals were reported when at least five studies contributed [17]. Compatible Class A results formed primary models; Class B results were added only in A + B sensitivity models. Inference emphasized effect magnitude, precision, consistency, and certainty rather than statistical significance alone.

### 2.9. Crossover, Factorial, and Multi-Arm Trials

Crossover trials were analysed as paired comparisons targeting the within-participant end-of-period difference, with each participant counted once. Reported paired effects and variances were used directly when available. When paired variance was unavailable, the within-participant correlation was assumed to be r = 0.50 for the primary Class B sensitivity estimate and varied to 0.25 and 0.75 in sensitivity analyses; such correlation-dependent estimates did not enter Class A primary models. A washout of at least 4 weeks was prespecified as a conservative design threshold to reduce the plausibility of carryover between randomized treatment periods. This threshold was used as an analytic safeguard rather than as a claim that 4 weeks represents a universal pharmacokinetic washout period across all CoQ10 doses and formulations. Crossover results with shorter, absent, or unclear washout were therefore routed to Class B sensitivity or standalone analysis when otherwise quantitatively recoverable.

For factorial trials, a CoQ10 main effect was used only when the randomized contrast was isolatable, co-interventions were balanced, and no important interaction invalidated the marginal effect. For multi-arm trials, comparable CoQ10 arms were combined, or a prespecified clinically relevant contrast was selected, and shared controls were not reused within a meta-analysis. First-period crossover data were used only when unbiased and separately available.

### 2.10. Missing Data, Digitization, and Statistical Conversions

When numerical data were incomplete, all available reports, supplements, protocols, and registrations were checked, and authors were contacted with one reminder after approximately 2 weeks. Standard errors, confidence intervals, exact test statistics, and exact *p* values were converted using conventional relationships only when the tested contrast and degrees of freedom were unambiguous; non-exact significance statements were not converted. When change-score variance was unavailable, eligible post-intervention comparisons were preferred rather than reconstructing change variance.

Means and SDs were estimated from medians and interquartile ranges using Wan and Luo methods [12,13] only when distributional assumptions were considered defensible; converted results were Class B. Graphical recovery required duplicate independent calibration and reconciliation, and no graph-only result passed all verification and comparability criteria. Units and effect directions were harmonized before pooling, while absolute oxygen uptake was not combined with relative-unit models without valid body-mass data. Original values, transformations, assumptions, and Class B inputs are documented in Supplementary Table S3.

### 2.11. Sensitivity and Influence Analyses

Robustness was assessed by comparing each Class A primary model with its compatible A + B model. Correlation-dependent crossover estimates used r = 0.50 with r = 0.25 and 0.75 as sensitivity bounds; VO_2_ analyses also excluded the no-washout study [18,19]. A separate high-risk-study exclusion analysis was prespecified but was not performed because the final Class A meta-analyses contained only 2–5 studies per outcome. Excluding high-risk trials would therefore have further reduced already sparse evidence bases and, for several outcomes, yielded unstable or non-informative pooled estimates. This was treated as a documented deviation from the planned analysis rather than replaced by an unplanned confirmatory model. Risk-of-bias concerns were instead incorporated into GRADE, and individual-study influence was examined using leave-one-out analyses when at least three studies were available. Leave-one-out analyses were conducted for A and A + B models with at least three studies, recording changes in pooled effects, 95% CIs, τ^2^, and I^2^; full results are in Supplementary Table S4.

The prespecified clinical-versus-nonclinical VO_2_ subgroup analysis was not performed because only five Class A studies were available, below the six-study threshold. The planned age comparison for 6-min walking distances was also infeasible because of limited study count and confounding with clinical population. Meta-regression was not undertaken because no outcome contained 10 comparable studies.

### 2.12. Reporting Bias and Certainty of Evidence

Funnel plots and regression-based asymmetry tests were planned only for outcomes with at least 10 comparable studies and meaningful size variation; no outcome met this threshold. Trim-and-fill was not used. Risk of bias due to missing evidence was assessed qualitatively at the meta-analysis-result level using ROB-ME-informed principles [20]. For each meta-analytic outcome, we considered whether eligible outcome results were known to have been measured but were unavailable for quantitative synthesis, whether trial protocols or registrations suggested selective non-reporting, and whether linked reports, reference-list checks, or author correspondence indicated additional unreported eligible results. Downgrading for publication bias was considered when these signals collectively suggested that missing evidence was plausibly related to the direction or magnitude of the observed effect.

GRADE was applied to seven key Class A outcomes: relative VO_2_peak/VO_2_max, 6-min walking distance, exercise duration, handgrip strength, lower-limb strength, chair-rise performance, and muscle/lean-tissue quantity [21]; maximum workload received a supplementary rating. Randomized evidence started at high certainty and could be downgraded for risk of bias, inconsistency, indirectness, imprecision, or publication bias. The prespecified target population encompassed adult study populations with a study-level mean or median age ≥ 40 years across both nonclinical and clinical settings. Indirectness was judged at the outcome level against this prespecified review question. Diagnosis alone was not considered sufficient for downgrading when the intervention, comparator, and underlying outcome construct remained clinically comparable. A downgrade was considered when the contributing evidence materially departed from the prespecified population, intervention, comparator, or outcome construct. Clinical heterogeneity within the eligible population was nevertheless considered when interpreting the applicability of pooled estimates to specific patient groups. Imprecision considered CI width, total information, and whether intervals spanned materially different interpretations. Formal GRADE ratings applied only to Class A primary evidence; A + B models were sensitivity analyses and were not rated.

### 2.13. Software and Reproducibility

Analyses were conducted in R 4.6.1 on Windows 11, primarily using metafor 5.0–1 for effect-size calculation, random-effects modelling, Hartung–Knapp inference, heterogeneity, correlation scenarios, and leave-one-out analyses. RoB 2 and GRADE judgments were stored in structured outcome-level files and were not inferred automatically from single statistics or plots. The locked dataset retained original values, transformations, evidence classes, assumptions, washout status, and analytic routes under stable identifiers; scripts generated the reported models, tables, and figures from these data. The reproducibility package contains the protocol and amendments, original database exports, executed searches, the known-item recovery audit, screening decisions, extraction dictionary, locked dataset, conversion records, scripts, source data, session information, and generated outputs and is available from the corresponding author on reasonable request.

## 3. Results

### 3.1. Study Selection

The database searches identified 2633 records, comprising 1190 records from PubMed, 268 from Embase, 541 from Scopus, 351 from Web of Science and 283 from CENTRAL. No records were removed by automation tools or for reasons other than duplication. After removal of 772 duplicate records, 1861 unique records underwent title and abstract screening, of which 1827 were excluded because they did not meet the prespecified eligibility criteria. In the post-hoc known-item audit, PubMed retrieved 32/33 final included reports, Embase 19/33, Web of Science 28/33, Scopus 29/33, and CENTRAL 31/33. The union of the five original exports retrieved all 33 final included reports (100%), although no single database was complete.

Thirty-four reports were sought for retrieval, all of which were successfully obtained and assessed in full text. One report, by Ramezani et al. [22], was excluded because it evaluated global neurological impairment and disability after acute ischaemic stroke but did not report a prespecified performance-based, physiological, or disease-specific motor outcome that directly measured muscle health or physical capacity. Consequently, 33 reports representing 33 eligible randomized studies were included in the systematic review. The complete study-selection process is presented in Figure 1.

### 3.2. Characteristics of Included Studies

The 33 randomized studies included were published between 1985 and 2026 and randomized a total of 3317 participants. Individual study sample sizes ranged from 12 to 609 randomized participants, although the number of participants available for eligible outcome analyses ranged from 7 to 609 because some crossover studies reported only complete-case data or separately extractable age-eligible subgroups. Twenty-three studies used parallel-group designs, including multi-arm and factorial trials from which an isolated CoQ10 comparison could be obtained, whereas 10 used randomized crossover designs. The studies were conducted across Europe, North America, Asia, and Australia and included both single-country and multinational trials.

The study populations were clinically heterogeneous. Six studies enrolled healthy or otherwise nonclinical participants, 10 enrolled participants with cardiovascular disease, four investigated statin-treated or statin-intolerant populations, seven enrolled participants with neurological or movement disorders, and six examined other clinical populations, including chronic kidney disease, maintenance haemodialysis, chronic obstructive pulmonary disease, mitochondrial cytopathy, Gulf War illness, and post-polio syndrome. Mean or median participant ages in the overall study samples generally ranged from approximately 40 to 75 years; one mixed-age crossover trial contributed only its separately reported subgroup aged 60–74 years.

CoQ10 was administered as conventional ubiquinone or CoQ10, ubiquinol, or an enhanced-bioavailability formulation, including phytosome, gel, nanoparticulate, and other solubilized preparations. Fixed daily doses ranged from 60 to 2400 mg/day, while one study used a weight-based dose of 5 mg/kg/day. Intervention periods ranged from 17 days to 60 months; in crossover trials, this range refers to the duration of each randomized treatment period. Most studies used a matched placebo comparator. Other eligible comparisons included no supplementation or an isolated no-CoQ10 factorial contrast. Several studies administered exercise training, statin treatment or standard disease-specific therapy equally to both randomized groups.

Eligible outcomes included aerobic capacity, walking capacity, exercise duration, maximum workload, strength, chair-rise performance, muscle quantity, and disease-specific clinician-rated motor function. Under the mutually exclusive A > B > C hierarchy, 19 studies were Class A, 10 Class B, and four Class C. Twelve Class A studies contributed to primary pooling, and seven were standalone only. Nine Class B studies contributed to sensitivity models, with Deichmann also providing a standalone result, and Moghadam was standalone only. Kuhlman was counted once in Class A despite additional Class C graph-only outcomes. Outcome-level routing remained authoritative (Table 1; Supplementary Tables S1–S3).

### 3.3. Risk of Bias

Risk-of-bias assessments were completed for all 33 included randomized trials using the design-appropriate RoB 2 tool. Overall, two trials (6.1%) were judged to be at low risk of bias, 16 (48.5%) raised some concerns, and 15 (45.5%) were judged to be at high risk of bias. The distribution of domain-level and overall judgments is presented in Figure 2.

For the randomization process, nine trials (27.3%) were low risk and 24 (72.7%) raised some concerns, mainly because sequence generation or allocation concealment was underreported. Dedicated Domain S was applied only to the 10 randomized crossover trials: two (20.0%) were low risk, four (40.0%) raised some concerns, and four (40.0%) were high risk. High Domain S judgments primarily reflected consecutive treatment periods without an adequate clearance interval and a plausible risk of residual CoQ10 or training effects. For deviations from intended interventions, 32 trials (97.0%) were low risk and one (3.0%) raised some concerns; no trial was high risk in Domain 2.

For missing outcome data, 20 trials (60.6%) were low risk, 12 (36.4%) raised some concerns, and one (3.0%) was high risk. All 33 trials were low risk for outcome measurement because outcomes were objectively measured or used standardized, validated performance, or motor-function assessments.

For selective reporting, 10 trials (30.3%) were low risk, 19 (57.6%) raised some concerns, and four (12.1%) were high risk. Concerns reflected unavailable prospective protocols, multiple eligible outcomes or time points, and occasionally inconsistent reporting. Overall high risk arose from a high-risk domain or accumulated concerns. Study-level judgments are provided in Supplementary Figure S8 and the completed RoB 2 audit workbooks.

### 3.4. Aerobic Capacity

The Class A analysis included five studies and 172 participants. The pooled relative VO_2_peak/VO_2_max effect was MD 0.73 mL·kg^−1^·min^−1^ (95% CI −0.07 to 1.54; *p* = 0.065), with τ^2^ = 0, I^2^ = 0%, and a prediction interval of −0.07 to 1.54. The estimate was small and compatible with little or no effect.

Adding three Class B studies yielded eight studies and 219 participants. The A + B estimate was MD 0.83 mL·kg^−1^·min^−1^ (95% CI 0.09 to 1.57; *p* = 0.033), with τ^2^ < 0.01, I^2^ < 0.01%, and a prediction interval of 0.09 to 1.57. The 0.09-unit change supported directional stability (Figure 3; Supplementary Figure S3).

The Class A evidence was low certainty because of risk of bias and imprecision. The A + B model was not separately graded because it included assumption-dependent estimates; it supported robustness but did not replace the Class A conclusion (Table 2).

GRADE downgrading reasons: a, serious risk of bias; b, very serious risk of bias; c, serious inconsistency; d, serious imprecision; e, very serious imprecision. CI, confidence interval; MD, mean difference. Hedges’ g is expressed in standard-deviation units. No outcome met the prespecified criterion for serious indirectness. Contributing populations were within the broad prespecified target population and pooled outcomes were considered comparable at the construct level; however, clinical heterogeneity limits subgroup-specific applicability.

### 3.5. Physical Capacity

For 6-min walking distance, four Class A studies (266 participants) yielded MD 34.58 m (95% CI −22.56 to 91.72; *p* = 0.150; τ^2^ = 1046.40; I^2^ = 81.7%). Adding two Class B studies produced MD 23.92 m across six studies (303 participants; 95% CI −13.01 to 60.85; *p* = 0.157; τ^2^ = 919.33; I^2^ = 77.3%), with a prediction interval of −62.33 to 110.17 m. Direction remained positive, but certainty was very low and both models were highly uncertain.

For exercise duration, two Class A studies (81 participants) produced Hedges’ g = 0.38 (95% CI −1.26 to 2.03; *p* = 0.207; τ^2^ = 0; I^2^ = 0%). Adding three Class B studies gave g = 0.17 across five studies (114 participants; 95% CI −0.40 to 0.74; *p* = 0.461; τ^2^ = 0.08; I^2^ = 44.9%), with a prediction interval of −0.81 to 1.15. Correlation scenarios yielded g = 0.13–0.21, all with CIs crossing the null. Class A certainty was very low.

Maximum workload was assessed in two Class A studies (63 participants), yielding MD 8.34 W (95% CI −220.71 to 237.39; *p* = 0.724; τ^2^ = 347.95; I^2^ = 50.8%). Adding two Class B studies produced MD 12.69 W across four studies (153 participants; 95% CI −10.84 to 36.22; *p* = 0.185; τ^2^ = 144.58; I^2^ = 82.5%). Class A certainty was very low.

Adding Class B evidence did not reverse any physical-capacity estimate, but every CI included the null. Class A certainty was very low for 6-min walking distance, exercise duration, and maximum workload (Figure 4; Table 2).

### 3.6. Muscle Health

For handgrip strength, four Class A studies (155 participants) yielded MD 1.85 kg (95% CI −2.08 to 5.79; *p* = 0.230; τ^2^ = 5.94; I^2^ = 89.6%). No compatible Class B handgrip result was available; Glover 2010 measured forearm torque in N·m and remained standalone. Class A certainty was very low.

Lower-limb strength in two Class A studies (57 participants) yielded MD 5.64 N·m (95% CI −222.12 to 233.40; *p* = 0.806; τ^2^ = 337.83; I^2^ = 52.1%). Adding one Class B study produced MD 1.07 N·m across three studies (71 participants; 95% CI −57.28 to 59.42; *p* = 0.944; τ^2^ = 225.29; I^2^ = 34.2%). Class A certainty was very low.

Chair-rise performance was pooled as Hedges’ g, with positive values indicating better performance. Three Class A studies (138 participants) yielded g = 1.16 (95% CI −2.45 to 4.76; *p* = 0.301; τ^2^ = 1.99; I^2^ = 94.5%). Adding one Class B study produced g = 0.76 across four studies (152 participants; 95% CI −1.55 to 3.07; *p* = 0.373; τ^2^ = 1.96; I^2^ = 93.3%). Class A certainty was very low.

For muscle/lean-tissue quantity, four Class A studies (120 participants) yielded g = 0.10 (95% CI −0.26 to 0.47; *p* = 0.435; τ^2^ = 0; I^2^ = 0%). Adding one Class B crossover study produced g = 0.06 across five studies (148 participants; 95% CI −0.15 to 0.27; *p* = 0.472; I^2^ = 0%), with the same prediction interval. Correlation scenarios yielded g = 0.04–0.07. Class A certainty was low.

All four muscle-health estimates were directionally positive, but every CI included the null. Strength and chair-rise findings were especially uncertain; muscle quantity was consistent but imprecise. Distinct constructs and metrics were not combined into one overall effect (Figure 5; Table 2).

### 3.7. Disease-Specific, Standalone, and Narrative Synthesis

Eight trials reported extractable prespecified outcomes that were not represented in the principal pooled physical-performance or muscle-health models: seven used disease-specific clinician-rated motor or functional scales and one used a capped walking time-to-exhaustion test. These outcomes were reported separately because their disease context, scale meaning, follow-up, or measurement properties precluded clinically appropriate pooling across disorders or with the principal objective-performance models.

Parkinson-disease findings were mixed. Beal et al. [25] found little or no separation from placebo on UPDRS Part III. Müller et al. [46] likewise reported no significant between-group benefit in UPDRS Part III despite modest improvements in total UPDRS and colour-vision performance, whereas Yoritaka et al. [53] reported directionally favourable but imprecise differences at 48 and 96 weeks. In Huntington disease, both CARE-HD [37] and 2CARE [42] produced confidence intervals compatible with little or no benefit on UHDRS motor or Total Functional Capacity outcomes. Mitsui et al. [43] reported a modest benefit on UMSARS Part II in multiple system atrophy at 48 weeks (MD −1.70 points, 95% CI −3.20 to −0.20), while the 10 m walk-time estimate was imprecise. Stamelou et al. [51] reported directionally favourable 6-week changes in progressive supranuclear palsy, but only 20 participants provided follow-up data. These neurological findings were therefore retained as disease-specific standalone evidence.

In chronic obstructive pulmonary disease, Moghadam et al. [44] reported greater improvement in capped walking time to exhaustion with CoQ10. Median-to-mean conversion yielded an approximate between-group difference of 2.00 min (95% CI 1.68 to 2.32), but the 6-min ceiling and distributional conversion made the estimate assumption-dependent and non-exchangeable with open-ended exercise-duration tests; it was therefore retained as B-standalone evidence.

Four additional randomized trials remained narrative only because no defensible effect estimate and variance could be reconstructed. Berman et al. and Mortensen et al. reported 6-min walking outcomes in heart failure without sufficient compatible variance or follow-up data; Golomb et al. reported a ceiling-limited lower-extremity composite that was not exchangeable with continuous performance outcomes; and Permanetter et al. reported maximum workload from a crossover trial with unresolved washout, sequence, and variance limitations. Their narrative disposition and reasons for non-pooling are summarized in Table 3.

### 3.8. Sensitivity and Influence Analyses

Adding compatible Class B evidence did not reverse any pooled effect direction. Relative VO_2_peak/VO_2_max changed from MD 0.73 to 0.83 mL·kg^−1^·min^−1^, with negligible heterogeneity. Other A + B estimates were directionally consistent with Class A estimates, but their CIs included the null. Handgrip had no compatible Class B result. Complete comparisons are in Supplementary Table S4.

For relative VO_2_peak/VO_2_max, r = 0.25 and 0.75 produced MDs of 0.80 (95% CI 0.11 to 1.50) and 0.88 mL·kg^−1^·min^−1^ (95% CI 0.07 to 1.69). Excluding the no-washout study yielded 0.67 (95% CI −0.08 to 1.42). Omitting Deichmann 2012 or Laaksonen 1995 yielded 0.84 (95% CI −0.07 to 1.74) and 0.89 (95% CI 0.25 to 1.53), respectively. Direction remained positive, but precision varied.

Leave-one-out VO_2_ estimates remained positive, although several A + B CIs crossed the null. Removing Bodea 2025 reduced 6-min walking I^2^ from 81.7% to 5.3% for Class A and from 77.3% to 21.4% for A + B. Thus, potentially influential high-risk studies were identified transparently through study-level influence analyses rather than through a separate exclusion model that would have left several outcome-specific evidence bases too sparse for stable inference. Removing Belardinelli 2006 reduced maximum-workload I^2^ from 82.5% to approximately 0%. Handgrip was sensitive to Fogacci 2024, whereas exercise duration, lower-limb strength, and chair rise remained imprecise. Muscle-quantity estimates were stable with I^2^ = 0%. Complete results are in Supplementary Table S4 and Figures S6 and S7.

No efficacy subgroup analysis was conducted because the five-study Class A model did not meet the six-study threshold. Age subgroup and meta-regression analyses were also not performed because feasibility and comparability criteria were unmet.

### 3.9. Certainty of Evidence

No Class A primary body of evidence was of high or moderate certainty. Certainty was low for relative VO_2_peak/VO_2_max and muscle/lean-tissue quantity, and very low for 6-min walking distances, exercise duration, handgrip strength, lower-limb strength, and chair-rise performance (Table 2). A + B models were not separately graded.

All seven outcomes were downgraded for risk of bias and imprecision. Risk of bias was very serious for walking distance and exercise duration, and serious otherwise. Imprecision was very serious for exercise duration and lower-limb strength. Walking distance, handgrip, lower-limb strength, and chair rise were also downgraded for inconsistency. No Class A outcome was downgraded for serious indirectness. The review question deliberately encompassed adult populations with a study-level mean or median age of ≥40 years across both nonclinical and clinical settings, and quantitative synthesis was restricted to outcomes judged to represent sufficiently comparable constructs. Accordingly, variation in underlying diagnosis was considered clinical heterogeneity within the prespecified target population rather than indirectness by itself. Disease-specific motor outcomes were not pooled across neurological disorders.

No Class A body of evidence was downgraded for publication bias. Across the primary outcomes, the qualitative ROB-ME-informed assessment did not identify a consistent pattern of known missing eligible results, protocol or registry discrepancies, or selective non-reporting that was judged likely to materially distort the pooled effect estimates. Eligible but non-quantifiable results were retained in the review and reported narratively rather than being excluded from consideration. Because no meta-analysis contained at least 10 comparable studies, formal funnel-plot or regression-based asymmetry analyses were not performed. Accordingly, the absence of a GRADE downgrade should not be interpreted as evidence that publication or selective-reporting bias was absent. A + B models preserved direction but did not change formal GRADE ratings. Maximum workload was supportive, very-low-certainty evidence and was excluded from the principal Summary of Findings table.

## 4. Discussion

### 4.1. Principal Findings

This systematic review of 33 randomized studies involving 3317 participants did not establish a clinically meaningful benefit of CoQ10 supplementation for muscle health or physical capacity. Relative VO_2_peak/VO_2_max was the pooled outcome with the least detected statistical heterogeneity, but it should not be interpreted as convincing evidence of benefit. The Class A estimate was small (0.73 mL·kg^−1^·min^−1^), based on only five studies, and imprecise, with a confidence interval that included no effect; the evidence was of low certainty. With so few studies, I^2^ = 0% does not demonstrate that the underlying effects are homogeneous. This result should therefore be interpreted as an imprecise directional estimate rather than evidence of a consistent or clinically meaningful benefit.

Effects on walking distance, exercise duration, maximum workload, handgrip strength, lower-limb strength, chair-rise performance, and muscle/lean-tissue quantity also remained uncertain, but the reasons differed across domains. For 6-min walking distances, handgrip strength, and chair-rise performance, wide confidence intervals, substantial heterogeneity, and risk-of-bias concerns limited interpretation. Exercise duration, maximum workload, and lower-limb strength were supported by particularly sparse evidence and could not distinguish benefit, no effect, or harm with adequate precision. Muscle/lean-tissue quantity had a point estimate close to the null and little detected heterogeneity, making little or no average effect more plausible than for the other domains, although the small evidence base, imprecision, and low certainty still precluded a definitive conclusion.

Separating directly supported Class A evidence from statistically recovered Class B evidence affected interpretation at the domain level. For relative VO_2_peak/VO_2_max, adding Class B estimates moved the confidence interval marginally above the null, but the expanded model remained assumption-dependent and did not replace the low-certainty Class A conclusion. For walking distance, exercise duration, lower-limb strength, chair-rise performance, and muscle/lean-tissue quantity, A + B models did not materially change the overall interpretation; no compatible Class B handgrip estimate was available. The pooled values are averages across clinically diverse populations and should not be extrapolated as uniform effects in trained adults, frail older people with sarcopenia, or patients with heart failure, chronic kidney disease, statin-associated symptoms, or neurological disorders.

### 4.2. Interpretation and Potential Mechanisms

The directionally favourable but inconclusive aerobic-capacity estimate is biologically plausible because CoQ functions as an electron carrier in the mitochondrial respiratory chain and contributes to cellular redox regulation and membrane antioxidant defence [5,54]. A recent meta-analysis of randomized trials reported an improvement in flow-mediated dilation following CoQ10 supplementation, suggesting a possible vascular contribution to peripheral oxygen delivery [55]. However, such vascular or biochemical effects do not establish a corresponding improvement in skeletal-muscle function or exercise performance, and mechanistic plausibility should not be equated with demonstrated tissue-level action. In robust older adults, 12 weeks of supplementation markedly increased plasma CoQ10 without increasing skeletal-muscle or mitochondrial CoQ10, improving mitochondrial respiration, or changing VO_2_max [49]. This biochemical-to-functional translation gap may explain why circulating responsiveness can coexist with small or absent performance effects.

The pattern of an inconclusive aerobic-capacity estimate without convincing gains in muscle quantity or strength could reflect systemic cardiovascular or endothelial effects rather than skeletal-muscle hypertrophy, although the included trials did not permit formal mediation analysis. Population context may also influence responsiveness. A randomized crossover trial in chronic kidney disease found no improvement in physical endurance following CoQ10 supplementation [23], whereas a trial in older adults with statin-associated asthenia reported improvements in several performance tests [31]. These contrasting observations raise, but do not prove, the hypothesis that baseline CoQ10 status, disease-related energetic limitation, formulation, treatment duration, tissue bioavailability, and concomitant exercise modify the functional response to supplementation.

### 4.3. Relation to Previous Evidence

The present findings help to resolve, rather than eliminate, earlier disagreement. A 2003 systematic review identified modest improvements in exercise capacity in six studies but no effect in five others [56]. More recently, Deng et al. concluded that CoQ10 reliably raises circulating concentrations but produces, at most, modest and context-dependent improvements in exercise performance among healthy adults, with low-to-very-low certainty [8]. Talebi et al. reported reductions in biomarkers of exercise-induced muscle damage and oxidative stress [7]; however, biomarker improvement does not necessarily translate into better strength, mobility, or aerobic capacity.

In heart failure, a Cochrane review found that the evidence for exercise-capacity improvement was inconclusive and downgraded the outcome primarily because of risk of bias and imprecision [57], closely paralleling the uncertainty observed in the present analysis of 6-min walking distance. Evidence concerning statin-associated muscle symptoms is similarly conflicting: one meta-analysis reported improvements in pain, weakness, cramps, and fatigue [58], whereas another found no benefit for myalgia or adherence to statin therapy [59]. Importantly, subjective muscle symptoms are not interchangeable with objectively measured strength, muscle quantity, or physical performance.

The present review extends previous syntheses by focusing on adult populations with a study-level mean or median age ≥ 40 years, separating clinically distinct outcome domains and distinguishing directly usable results from estimates requiring paired-correlation or distributional assumptions. Compatible recovered estimates were used only in A + B sensitivity models, while non-exchangeable A or B results were reported separately and unverified graph-only results received no quantitative weight. This framework shows that a favourable direction can remain stable under reasonable recovery assumptions without becoming high-certainty evidence. Separating robustness from certainty is therefore a central methodological and interpretive contribution of the review.

### 4.4. Clinical and Research Implications

The pooled aerobic-capacity effect should be interpreted cautiously. In a randomized trial involving patients with heart failure with preserved ejection fraction, 2.5 mL·kg^−1^·min^−1^ was prespecified as the minimum clinically important difference for peak oxygen uptake [60], which is larger than the point estimate observed in the present review. However, this threshold cannot be generalized across all populations included here. Published estimates of clinically meaningful change in the 6-min walking distance also vary according to the clinical population, outcome anchor, and follow-up interval. An earlier systematic review in chronic heart failure proposed a change of approximately 45 m [61], whereas a more recent anchor-based analysis in patients with heart failure with reduced ejection fraction and iron deficiency estimated approximately 14–15 m for a small but clinically meaningful improvement [62]. The Class A estimate of 34.58 m therefore lies within the range of published heart-failure-specific thresholds. Nevertheless, its wide confidence interval encompassed possible worsening, little or no effect, and larger benefits, and neither threshold can be directly extrapolated to healthy trained adults, community-dwelling older adults, or patients with neurological disorders. The 6-min walking finding should therefore be regarded as clinically uncertain rather than definitively meaningful or non-meaningful. The clinically important threshold may differ between healthy trained participants, who have higher baseline fitness and different performance variability, and clinical cohorts, in whom baseline impairment, prognosis, and therapeutic goals differ; 2.5 mL·kg^−1^·min^−1^ should therefore be treated as a contextual benchmark rather than a universal cutoff.

The current evidence therefore does not support routine CoQ10 supplementation solely to improve physical capacity or muscle health. Future trials should be adequately powered, prospectively registered, and stratified according to clinical population and baseline CoQ10 status. They should use formulations with documented bioavailability; measure plasma and, where feasible, skeletal-muscle exposure; and prespecify standardized VO_2_peak/VO_2_max, 6-min walking distance, strength, chair-rise, and muscle-quantity outcomes. Exact group estimates, change-score variances, and within-participant correlations should be reported to prevent otherwise eligible crossover and repeated-measures data from becoming assumption-dependent. Factorial trials combining CoQ10 with exercise should also be designed to estimate whether supplementation provides an incremental benefit beyond exercise alone.

### 4.5. Strengths and Limitations

This review combined a reproducible five-database search with outcome-level evidence routing, structured RoB 2 assessments and a ROB-ME-informed appraisal, Hartung–Knapp random-effects models, leave-one-out analyses, and outcome-specific GRADE ratings. Separating directly supported Class A evidence from Class B estimates is an important methodological strength because it preserves potentially informative data without allowing unverifiable assumptions to determine the primary conclusion.

Several limitations remain. Outcome-specific meta-analyses contained few studies, preventing reliable meta-regression, subgroup analysis, dose-response modelling, and formal assessment of small-study effects. Accordingly, the ROB-ME-informed assessment was qualitative, and residual publication or selective-reporting bias cannot be excluded. This was a deliberately conservative choice because funnel plots and regression-based asymmetry tests can be unstable and misleading when based on fewer than 10 comparable studies. Search strategies were database-specific, and known-item recovery was lower for searches that used additional precision blocks, particularly Embase. Nevertheless, the union of the five original exports recovered all 33 final included reports, and the permissive dual-review screening rule advanced any uncertain or potentially eligible record to full-text assessment. This post-hoc audit strengthens confidence that the executed search set captured the known eligible evidence, but its benchmark was derived from the completed review and therefore could not identify eligible reports absent from both the benchmark and the database results. Residual retrieval bias from incomplete indexing, uninformative titles or abstracts, or non-indexed studies consequently cannot be excluded. Populations, formulations, doses, durations, co-interventions, and measurement protocols varied substantially. Although pooling was restricted to outcomes judged to represent sufficiently comparable clinical constructs, residual clinical heterogeneity remains because the same performance measure may reflect different physiological limitations across healthy, cardiovascular, metabolic, renal, and neuromuscular populations. Random-effects modelling addresses statistical heterogeneity but cannot eliminate these differences in clinical context. The relative VO_2_peak/VO_2_max signal was also disproportionately informed by studies of physically trained or athletic participants. Their baseline fitness, training adaptations, and physiological reserve differ from those of frail older adults with sarcopenia, who were not specifically represented as a target population. Accordingly, this aerobic-capacity signal should not be extrapolated directly to older sarcopenic populations. Therefore, pooled estimates should be interpreted as average effects for a shared outcome construct across the prespecified population rather than as evidence of an identical treatment effect across diagnostic groups. These differences did not warrant a GRADE downgrade for indirectness because they occurred within the prespecified broad target population and the pooled outcomes represented comparable constructs; nevertheless, they restrict the extent to which the average pooled effects can be assumed to apply uniformly to any specific clinical subgroup. Risk-of-bias concerns were frequent. The dedicated randomized-crossover RoB 2 variant was applied to all 10 crossover trials; however, older reports often incompletely described sequence allocation, period adjustment, or the rationale for washout duration. Four crossover trials were consequently judged to be at high risk in Domain S because an adequate clearance interval could not be established, and four raised some concerns. Use of the design-specific tool therefore improved characterization of period and carryover bias but could not remove uncertainty created by incomplete reporting. Confidence intervals were generally wide, and some A + B estimates required correlation or distributional assumptions. Unverified graph-only outcomes were not quantified, and incomplete reporting confined several eligible trials to narrative synthesis. Finally, the age criterion was based primarily on the study-level mean or median age rather than an individual-level minimum age. Some eligible studies therefore included participants younger than 40 years, and the available evidence did not permit reliable assessment of whether effects differed across middle-aged, older, and very old populations. Collectively, these limitations make the observed aerobic-capacity signal hypothesis-supporting rather than practice-changing.

## 5. Conclusions

Current randomized evidence does not establish that CoQ10 supplementation improves relative VO_2_peak/VO_2_max, walking capacity, exercise performance, muscle strength, chair-rise performance, or muscle/lean-tissue quantity in adult populations meeting the study-level mean or median age criterion of ≥40 years. The relative VO_2_peak/VO_2_max estimate was directionally favourable but small, imprecise, compatible with no benefit, and supported by low-certainty evidence; it should not be interpreted as a clinically meaningful effect. Disease-specific clinician-rated motor and functional outcomes were mixed and should not be interpreted as one transferable neurological effect. Adding assumption-dependent evidence generally preserved effect directions but did not increase the certainty of the primary evidence. Current evidence therefore does not support routine CoQ10 supplementation solely to improve muscle health or physical capacity. Future trials should be adequately powered and preregistered, use standardized performance-based, physiological, and clinician-rated motor-function outcomes, and report complete variance data. Randomized crossover trials should use a washout of at least 4 weeks when biologically appropriate, justify any alternative interval for the selected dose and formulation, and report paired analyses and within-participant correlations.

## Figures and Tables

**Figure 1:**
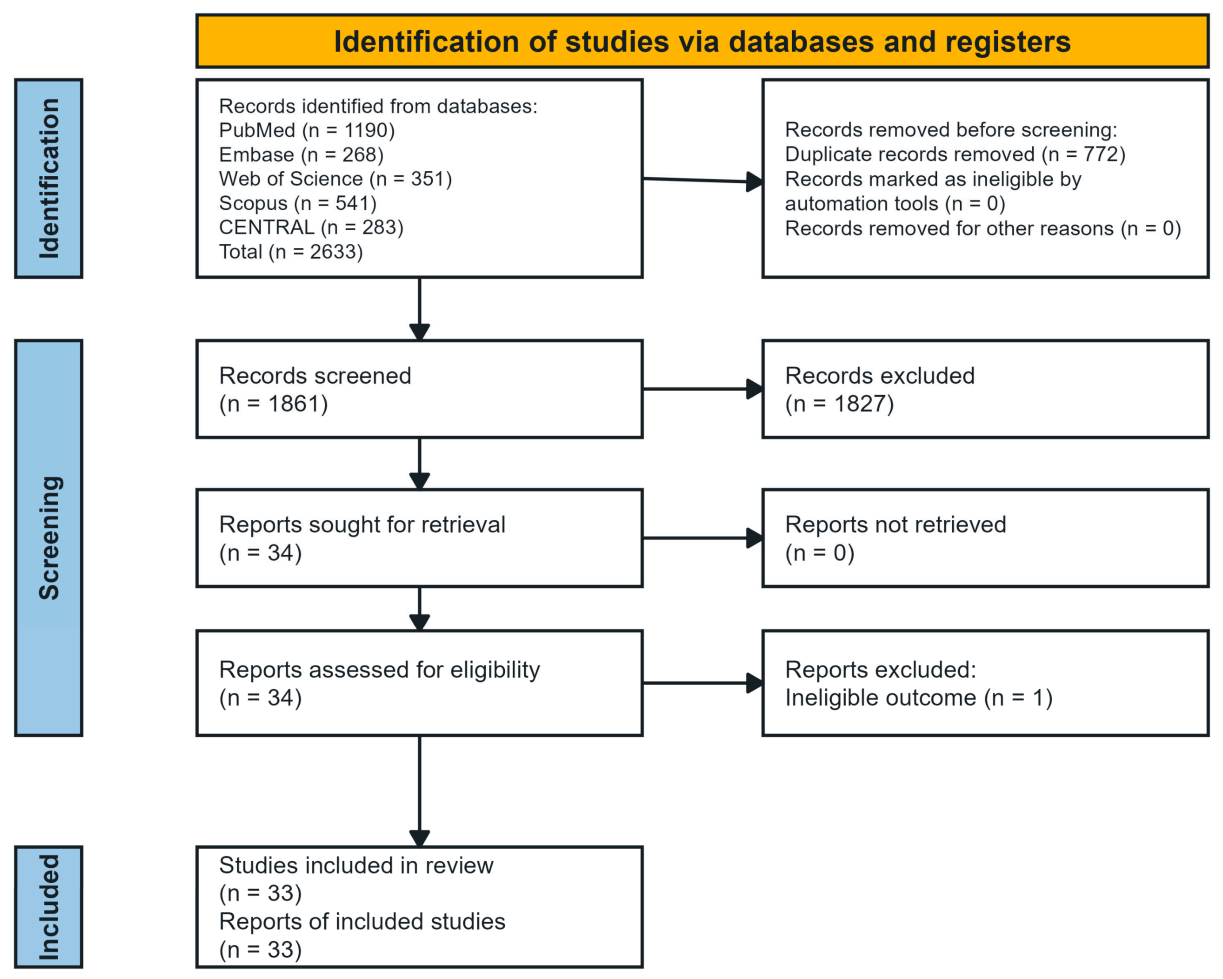
PRISMA 2020 flow diagram showing identification, screening, eligibility assessment, and inclusion of randomized trials evaluating oral CoQ10 and eligible physical-performance, muscle-health, or clinician-rated motor-function outcomes.

**Figure 2:**
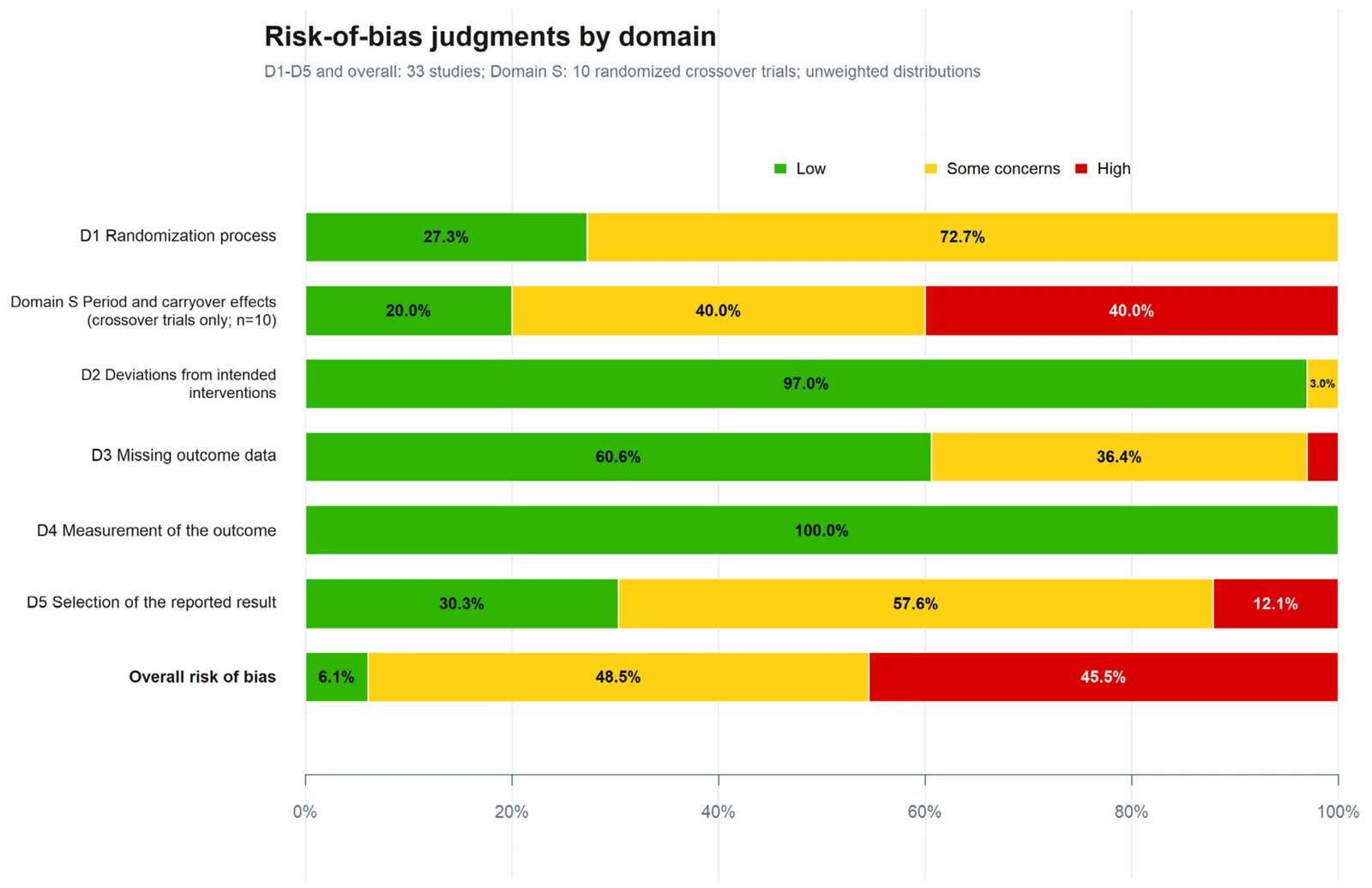
Summary of Cochrane RoB 2 judgments. D1–D5 and overall judgments include all 33 studies; Domain S (period and carryover effects) includes the 10 randomized crossover trials assessed with the dedicated crossover variant.

**Figure 3:**
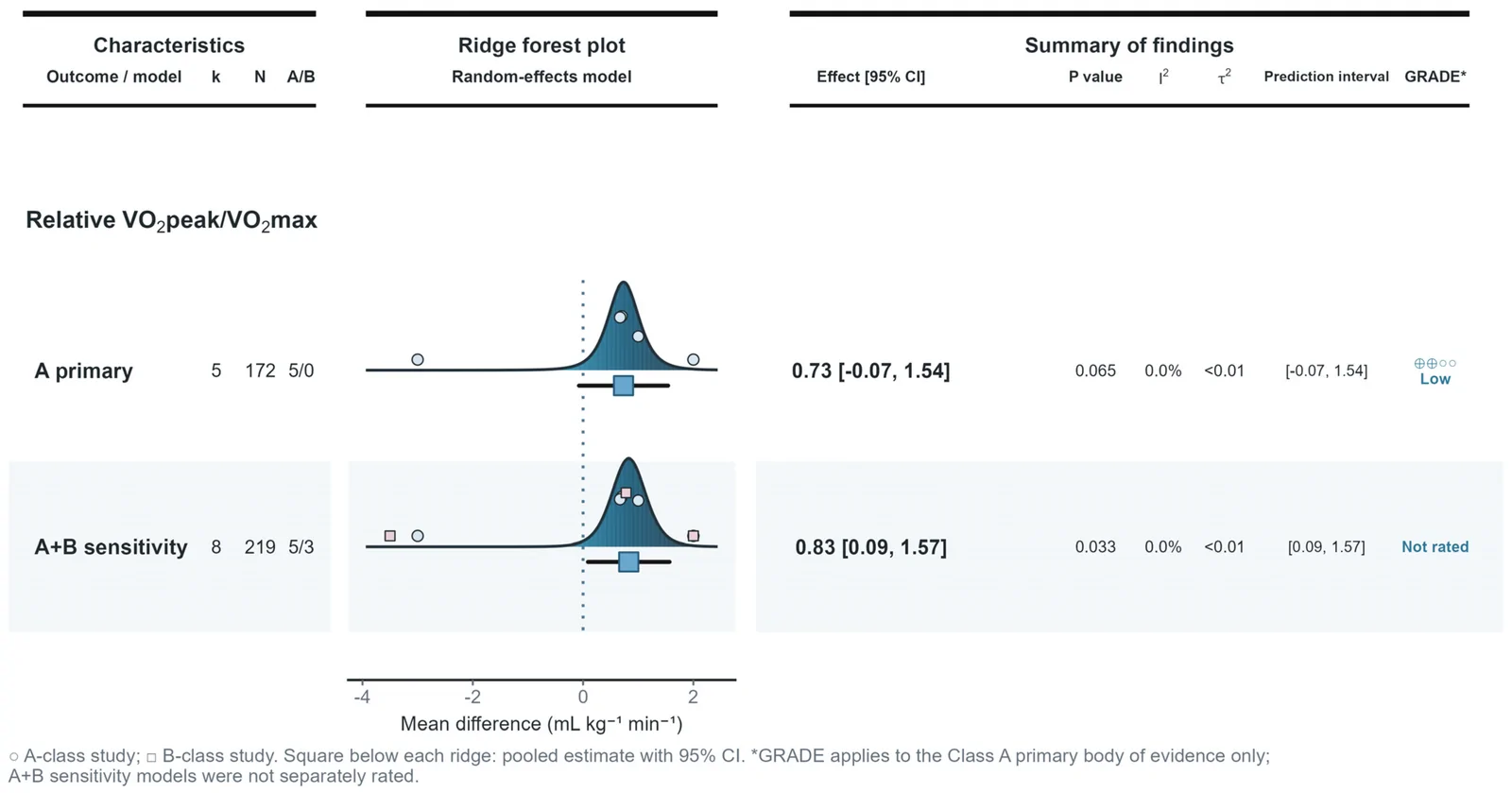
Effects of CoQ10 supplementation on relative VO_2_peak/VO_2_max. The figure contrasts the Class A primary model with the A + B sensitivity model and reports effect estimates, uncertainty, and heterogeneity. Formal GRADE certainty is shown only for the Class A primary body of evidence; the A + B row is marked “Not rated”.

**Figure 4:**
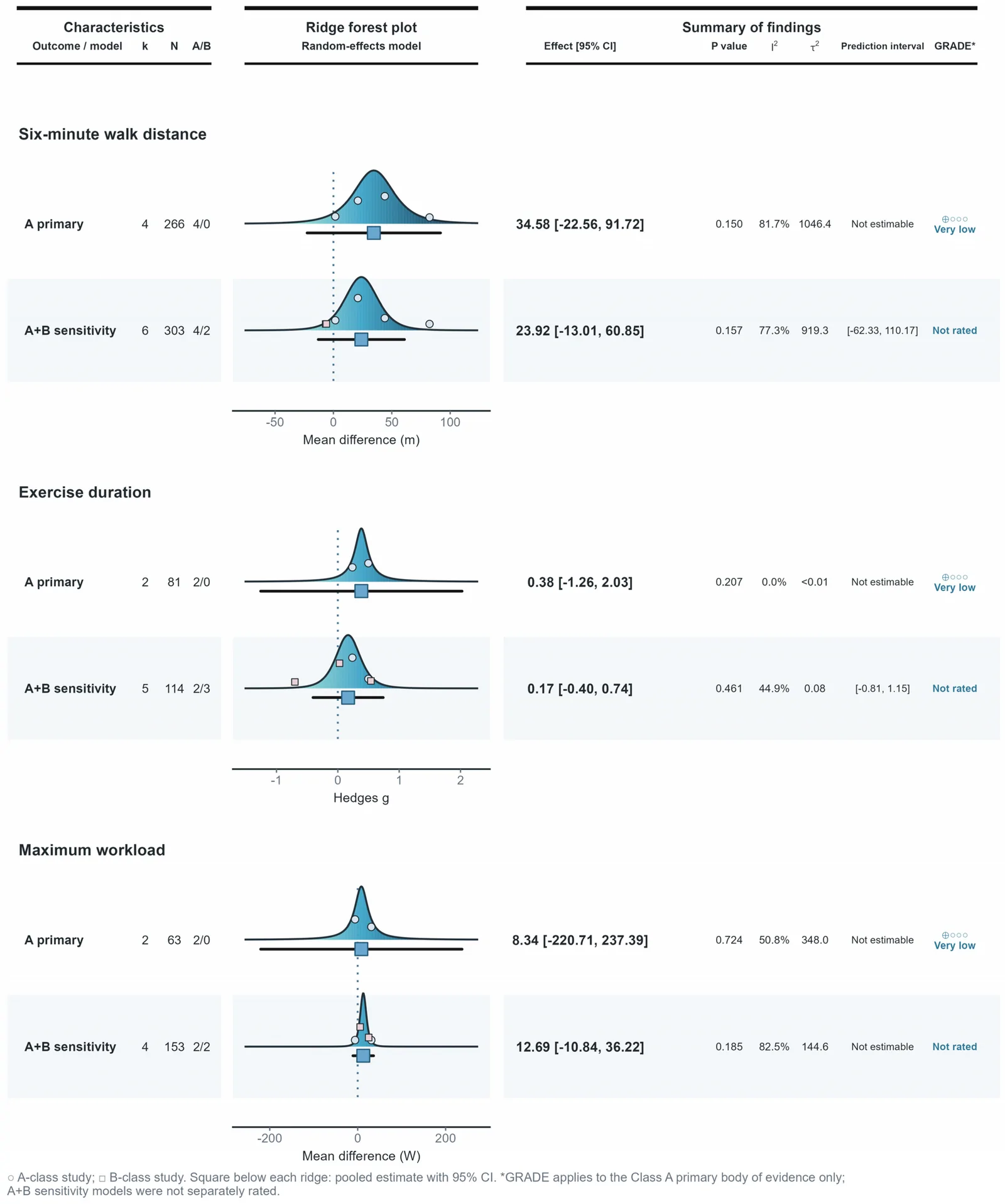
Effects of CoQ10 supplementation on physical−capacity outcomes, including 6-min walking distance, exercise duration, and maximum workload. Exercise duration is expressed as Hedges’ g; the other outcomes use mean differences in their stated units. Class A primary and A + B sensitivity models are shown with uncertainty and heterogeneity. GRADE certainty is shown only for the Class A primary body of evidence; A + B rows are marked “Not rated”.

**Figure 5:**
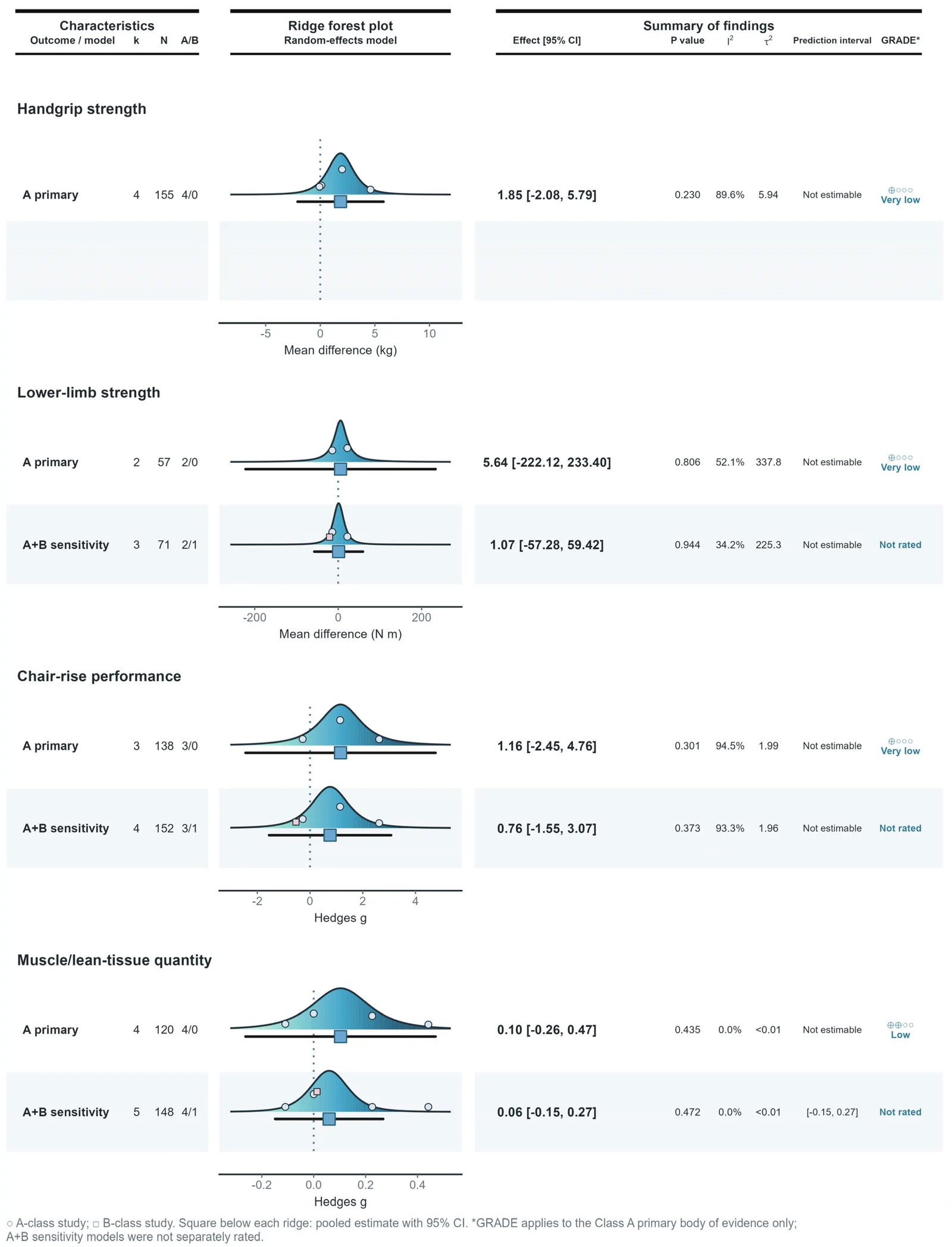
Effects of CoQ10 supplementation on muscle-health outcomes, including handgrip strength, lower-limb strength, chair-rise performance, and muscle/lean-tissue quantity. Chair−rise performance and muscle/lean−tissue quantity are expressed as Hedges’ g; strength outcomes use mean differences in their stated units. Class A primary and A + B sensitivity models are shown with uncertainty and heterogeneity. GRADE certainty is shown only for the Class A primary body of evidence; A + B rows are marked “Not rated”.

**Table 1:** Characteristics of included randomized trials. Each trial is counted once under its highest available evidence class using the hierarchy A > B > C; slash-separated labels preserve mixed outcome-level routes without double counting (definitions in Section 2.6). The n (R/A) column reports study-level randomized/analysed totals; meta-analytic N values are outcome-specific, and participants in crossover trials are counted once.

Study (Country)	Population	Design	n (R/A)	Age, y (Mean ± SD Unless Otherwise Stated)	CoQ10 Regimen	Comparator; Duration	Eligible Performance/ Motor Outcomes	Study-Level Route
Ahmadi 2023 [23] (USA)	Chronic kidney disease; eGFR < 60	Three-period double-blind crossover RCT	25/25	Overall: 61.0 ± 11.6	CoQ10 1200 mg/day	Placebo; 6 weeks per period	VO_2_peak; total work; test duration	A-pooled
Bagheri 2025 [24] (Iran)	Healthy sedentary adults aged 65–75	Parallel double-blind RCT; sex-stratified	38/38	CoQ10: 69 ± 4; placebo: 70 ± 4	CoQ10 100 mg/day	Matched placebo; 8 weeks	6MWD; chair-rise; grip; TUG; 25-ft walk; SLS; muscle mass	A-pooled
Beal 2014 [25] (QE3) (USA/Canada)	Early Parkinson disease	Multicentre three-arm parallel RCT	600/600	62.5	CoQ10 1200 or 2400 mg/day	Placebo; Mean follow-up 10.4 months; planned 16 months	UPDRS Part III motor score	A-standalone
Belardinelli 2006 [26] (Italy)	NYHA II-III chronic heart failure secondary to ischemic heart disease	Four-period randomized double-blind placebo-controlled crossover factorial trial	23/21	59 ± 9	CoQ10 100 mg three times daily (300 mg/day) (enhanced formulation)	Placebo; 4 weeks per phase	VO_2_peak; peak work rate	B-sensitivity
Berman 2004 [27] (Israel)	End-stage heart failure awaiting cardiac transplantation	Parallel randomized double-blind placebo-controlled trial	32/27	54.6 (range 40–67)	Ultrasome CoQ10 60 mg/day (enhanced formulation)	Placebo; 3 months	6MWD	C-narrative
Bloomer 2012 [28] (USA)	Exercise-trained adults aged 30–65; study mean age ≥ 40	Double-blind crossover RCT	17/15	42.7 ± 10.4	Ubiquinol 300 mg/day (ubiquinol)	Placebo; 4 weeks per period	Treadmill duration; sprint peak/mean power; total work	B-sensitivity
Bodea 2025 [29] (Romania)	NYHA II–III heart failure	Parallel double-blind RCT	120/120	CoQ10: 66.5 ± 12.8; placebo: 67.7 ± 14.3	CoQ10 60 mg twice daily (120 mg/day)	Identical placebo; 24 weeks	6MWD	A-pooled
Bonetti 2000 [30] (Italy)	Male leisure-time cyclists aged 30–50	Parallel randomized single-blind trial	28/23	CoQ10: 40.6 ± 6.0; placebo: 40.1 ± 7.4	CoQ10 100 mg/day	Placebo; 8 weeks	VO_2_peak; Wmax	A-pooled
Deichmann 2012 [18] (USA)	Athletes aged ≥50 taking stable statin doses	Double-blind crossover RCT	20/19	63.5 ± 8.2	CoQ10 200 mg/day (gel capsule)	Placebo; 6 weeks per period	VO_2_max change; anaerobic-threshold time; leg-extension repetitions	B-sensitivity/ B-standalone
Fogacci 2024 [31] (Italy)	Adults aged 65–80 with statin-associated asthenia	Single-centre parallel double-blind RCT	60/60	CoQ10: 74 ± 2; placebo: 73 ± 3	Ubiqsome 300 mg/day, providing CoQ10 60 mg/day (phytosome)	Matched placebo; 8 weeks	Handgrip; 1-min sit-to-stand; 2-min step	A-pooled
Glover 2010 [32] (Canada)	Adults with mitochondrial cytopathy	Double-blind crossover RCT	30/30	MELAS 48 ± 3; other diagnoses 56 ± 3	CoQ10 600 mg twice daily (1200 mg/day) (Qgel)	Soybean-oil placebo; 60 days per period	Maximal isometric forearm torque; lean body mass; fixed-workload cycling VO_2_	B-sensitivity
Gokbel 2016 [33] (Turkey)	Maintenance hemodialysis patients	Double-blind crossover RCT	28/23	46.6 ± 11.9	CoQ10 200 mg/day	Dextrose placebo; 12 weeks per period	6MWD; estimated VO_2_max	B-sensitivity
Golomb 2014 [34] (USA)	Veterans meeting criteria for Gulf War illness	Three-arm randomized double-blind placebo-controlled trial; first parallel phase used	46/42	100 mg: 48 ± 6.1; 300 mg: 50 ± 7.6; placebo: 44 ± 6.0	CoQ10 100 or 300 mg/day (softgel)	Identical placebo; 3.5 ± 0.5 months	Summary performance score	C-narrative
Hofman-Bang 1995 [35] (Sweden)	Stable chronic congestive heart failure	Multicentre double-blind crossover RCT	79/69	61 ± 10	CoQ10 100 mg/day	Matching placebo; 3 months per period	Wmax	B-sensitivity
Holloway 2014 [36] (United Kingdom)	Healthy adults undertaking a high-altitude trek	Parallel assessor-blinded RCT	23/23	CoQ10: 48.5 ± 14.9; control: 43.1 ± 15.5	CoQ10 300 mg/day	No supplement; 17 days	Lean body mass	A-pooled
Huntington Study Group 2001 [37] (CARE-HD) (USA/Canada)	Early Huntington disease	Multicentre 2 × 2 factorial RCT	347/347	~48	CoQ10 600 mg/day	No-CoQ10 factorial contrast; 30 months	UHDRS total motor; Total Functional Capacity	A-standalone
Kamikawa 1985 [38] (Japan)	Chronic stable angina pectoris	Randomized double-blind crossover trial after single-blind run-in	12/12	56 (range 45–66)	CoQ10 50 mg three times daily (150 mg/day)	Placebo; 4 weeks per period	Exercise duration; time to 1-mm ST depression	B-sensitivity
Keogh 2003 [39] (Australia)	NYHA II–III chronic heart failure	Parallel double-blind RCT	39/35	CoQ10: 62 ± 7; placebo: 61 ± 9	CoQ10 150 mg/day	Matched placebo; 12 weeks	6MWD; Naughton exercise time	A-pooled
Khatta 2000 [40] (USA)	NYHA III–IV congestive heart failure	Parallel double-blind RCT	55/46	64	CoQ10 200 mg/day	Placebo; 6 months	VO_2_peak; exercise duration	A-pooled
Kuhlman 2021/2022 [41] (Denmark)	Statin-naïve dyslipidaemic men aged 40–70	Three-arm parallel randomized double-blind RCT	32/26	CoQ10: 56 ± 8; placebo: 56 ± 9	Simvastatin 40 mg/day + CoQ10 400 mg/day (Ubiquinone)	Simvastatin 40 mg/day + placebo; 8 weeks	Knee-extension MVC; lean mass; grip; VO_2_max; Wmax	A-pooled/C
Laaksonen 1995 [19] (Finland)	Trained men; separately extractable older subgroup aged 60–74	Double-blind crossover RCT	19/7	63.8 (range 60–74)	Ubiquinone 120 mg/day (Ubiquinone)	Placebo; 6 weeks per period	VO_2_max; time to exhaustion	B-sensitivity
McGarry 2017 [42] (2CARE) (USA/Canada/Australia)	Early Huntington disease	Multicentre parallel double-blind RCT	609/609	CoQ10: 50.5 ± 11.9; placebo: 50.7 ± 11.6	CoQ10 2400 mg/day	Placebo; Up to 60 months	UHDRS total motor; Total Functional Capacity	A-standalone
Mitsui 2023 [43] (Japan)	Multiple system atrophy	13-centre parallel randomized double-blind RCT	139/129	CoQ10: 59.8 ± 8.5; placebo: 62.0 ± 8.3	Ubiquinol titrated to 1500 mg/day (Ubiquinol)	Placebo; 48 weeks	UMSARS Part II; 10-m walk time	A-standalone
Moghadam 2023 [44] (Iran)	Moderate-to-severe COPD	Parallel double-blind placebo-controlled RCT; registry confirms lottery allocation	90/90	CoQ10: 64.20 ± 11.38; placebo: 66.98 ± 12.16	CoQ10 120 mg/day	Identical placebo; 6 weeks	Walking time to exhaustion, capped at 6 min	B-standalone
Mortensen 2014 [45] (Q-SYMBIO) (Multinational)	Moderate-to-severe chronic heart failure	Multicentre parallel randomized double-blind placebo-controlled trial	420/420	62.3 ± 12 vs. 62.3 ± 11	CoQ10 100 mg three times daily (300 mg/day)	Placebo plus standard therapy; 16 weeks for 6MWD; 2 years overall	6MWD	C-narrative
Müller 2003 [46] (Germany)	Stable Parkinson disease	Single-centre parallel double-blind RCT	28/28	CoQ10: 66.21 ± 9.33; placebo: 64.36 ± 7.69	CoQ10 180 mg twice daily (360 mg/day)	Placebo; 4 weeks	UPDRS Part III	A-standalone
Permanetter 1992 [47] (Germany)	Idiopathic dilated cardiomyopathy (NYHA class I–III)	Randomized double-blind placebo-controlled crossover trial	25/24	54 ± 12 vs. 51 ± 8	Ubiquinone 33.3 mg three times daily (approximately 100 mg/day) (Ubiquinone)	Placebo; 4 months per period	Maximum workload	C-narrative
Pierce 2022 [48] (USA)	Heart failure with preserved ejection fraction	Four-arm parallel randomized double-blind RCT	153/139	69.2 ± 10.1	Ubiquinol 600 mg/day (Ubiquinol)	Double placebo; 12 weeks	6MWD	A-pooled
Schmücker 2026 [49] (Denmark)	Robust community-dwelling older adults	Parallel randomized double-blind placebo-controlled trial	40/40	CoQ10: 74 ± 4; placebo: 75 ± 4	Ubiquinone 400 mg/day (Ubiquinone)	Placebo; 12 weeks	VO_2_max; Wmax; lean mass; 30-s sit-to-stand	A-pooled
Skough 2008 [50] (Sweden)	Post-polio syndrome	Parallel randomized double-blind placebo-controlled pilot trial	14/14	Median—CoQ10: 71; placebo: 66	CoQ10 100 mg twice daily (200 mg/day)	Placebo; 12 weeks	Sit-stand-sit; TUG; 6MWD; knee strength; total work	B-sensitivity
Stamelou 2008 [51] (Germany)	Clinically probable progressive supranuclear palsy	Single-centre parallel double-blind RCT	21/20	Median—CoQ10: 63.5; placebo: 68.0	CoQ10 5 mg/kg/day (nanoparticulate)	Matched placebo; 6 weeks	PSP Rating Scale; UPDRS Part III	A-standalone
Taylor 2015 [52] (USA)	Adults with confirmed statin myalgia	Initial parallel randomized double-blind phase; later partial crossover	41/38	CoQ10: 58 ± 10; placebo: 60 ± 10	Simvastatin 20 mg/day + ubiquinol 600 mg/day (Ubiquinol)	Simvastatin 20 mg/day + placebo; 8 weeks after 2-week loading	VO_2_max; grip; knee and elbow strength	A-pooled
Yoritaka 2015 [53] (Japan)	Parkinson disease; wearing-off and non-levodopa cohorts	Two parallel double-blind RCT cohorts	64/48	Cohort A—CoQ10: 64.1; placebo: 61.5; cohort B—CoQ10: 59.8; placebo: 64.4	Ubiquinol 300 mg/day (Ubiquinol)	Placebo; 48 weeks (A); 96 weeks (B)	UPDRS Part III; total UPDRS	A-standalone

**Table 2:** Summary of quantitative findings and certainty of evidence for the Class A primary analyses.

Outcome	Evidence Base, k (N)	Pooled Effect (95% CI)	95% Prediction Interval	I^2^	Certainty (GRADE)	Interpretation
Relative VO_2_peak/VO_2_max	5 (172)	MD 0.73 (−0.07 to 1.54) mL·kg^−1^·min^−1^	−0.07 to 1.54 mL·kg^−1^·min^−1^	0.0%	Low (a, d)	Small, directionally favourable effect; the confidence interval includes little or no effect.
Six-minute walk distance	4 (266)	MD 34.58 (−22.56 to 91.72) m	Not estimated	81.7%	Very low (b, c, d)	Possible improvement, but the confidence interval is very wide, includes little or no effect, and heterogeneity is considerable.
Exercise duration	2 (81)	Hedges’ g 0.38 (−1.26 to 2.03) SD units	Not estimated	0.0%	Very low (b, e)	The standardized effect is highly uncertain because the confidence interval is extremely wide.
Handgrip strength	4 (155)	MD 1.85 (−2.08 to 5.79) kg	Not estimated	89.6%	Very low (a, c, d)	The effect is highly uncertain because the confidence interval crosses the null and heterogeneity is substantial.
Lower-limb strength	2 (57)	MD 5.64 (−222.12 to 233.40) N·m	Not estimated	52.1%	Very low (a, c, e)	The effect is highly uncertain; the confidence interval includes large effects in both directions.
Chair-rise performance	3 (138)	Hedges’ g 1.16 (−2.45 to 4.76) SD units	Not estimated	94.5%	Very low (a, c, d)	The pooled estimate favours CoQ10, but the confidence interval crosses the null and heterogeneity is substantial.
Muscle/ lean-tissue quantity	4 (120)	Hedges’ g 0.10 (−0.26 to 0.47) SD units	Not estimated	0.0%	Low (a, d)	No clear standardized effect was detected; the confidence interval includes effects in both directions.

**Table 3:** Narrative-only eligible studies and reason for non-pooling.

Study	Population and Design	Intervention and Comparator	Eligible Performance or Motor Outcome	Reported Result	Overall RoB 2	Reason Not Pooled
Berman 2004 [27]	End-stage heart failure awaiting transplantation; parallel double-blind RCT; 32 randomized and 27 completed	Enhanced-bioavailability CoQ10 60 mg/day vs. placebo; 3 months	6-min walking distance	Mean distance increased from 269.5 to 382.2 m with CoQ10 and decreased from 254 to 177 m with placebo; no compatible variance for the between-group effect was reported.	High	Group-specific SD, SE, or CI values were unavailable, and an inequality-reported *p*-value could not recover a defensible variance.
Golomb 2014 [34]	Veterans with Gulf War illness; first randomized parallel phase; 46 randomized	CoQ10 100 or 300 mg/day vs. placebo; approximately 3.5 months	Lower-extremity Summary Performance Score	Improvement occurred in 9/11 (82%) at 100 mg/day, 6/11 (55%) at 300 mg/day and 8/20 (40%) with placebo.	High	The 12-point composite combined chair rises, balance, and walking velocity and was converted to a binary improvement outcome because of ceiling effects; it was not compatible with the continuous outcome pools.
Mortensen 2014 [45] (Q-SYMBIO)	Moderate-to-severe chronic heart failure; multicentre double-blind parallel RCT; 420 randomized	CoQ10 300 mg/day vs. placebo; 16-week short-term assessment	Six-minute walk distance	Baseline distances were 287 ± 98 m and 286 ± 92 m in the CoQ10 and placebo groups, respectively. Both groups improved at 16 weeks, with no reported statistically significant between-group difference.	Some concerns	Compatible follow-up means, change-score variances, and a CI were not reported in the main publication; the treatment effect and its precision could not be reconstructed.
Permanetter 1992 [47]	Idiopathic dilated cardiomyopathy; two-sequence double-blind crossover RCT; 25 randomized	CoQ10 approximately 100 mg/day vs. placebo; 4 months per period	Maximum workload	Workload remained essentially unchanged in one sequence and showed only a marginal improvement in the other; no clear treatment effect was documented.	High	The outcome was graph-based, exact period means and paired variance were unavailable, no adequate washout was used, and baseline or sequence imbalance and possible carryover could not be resolved.

## Data Availability

All study-level and outcome-level data supporting the findings of this review are included in the article and its Supplementary Information. Extracted study characteristics, core quantitative data, assumption-dependent data and sensitivity-analysis results are reported in Supplementary Tables S1–S4, and the associated study-level, influence and risk-of-bias graphics are provided in Supplementary Figures S1–S8. No individual participant data were obtained; the source trial reports are cited in the reference list. The R scripts used for data processing, statistical synthesis, sensitivity and influence analyses, and figure generation are available from the corresponding author upon reasonable request.

## Supplementary Materials

The following supporting information can be downloaded at: https://www.mdpi.com/article/10.3390/antiox15101219/s1.
