# Supplementary material for: Effects of Coenzyme Q10 Supplementation on Muscle Health and Physical Capacity in Middle-Aged and Older Adults: A Systematic Review and Meta-Analysis of Randomized Controlled Trials
Source: Antioxidants (Basel). 2026 Sep 22;15(10):1219. doi: 10.3390/antiox15101219 (PMC13700822; doi:10.3390/antiox15101219)
Supplement: Supplementary file 1 [file antioxidants-15-01219-s001.zip › 01_Supplementary_Methods_and_Search_Strategies.pdf]

Supplementary Methods S1

Eligibility criteria and electronic search strategies

Effects of Coenzyme Q10 Supplementation on Muscle Health and Physical Capacity in Middle-Aged and Older Adults: A Systematic Review and Meta-Analysis of Randomized Controlled Trials

PROSPERO: CRD420261469564

S1. Eligibility criteria

Eligibility was defined using the PICOS framework. Oral ubiquinone (ubidecarenone), ubiquinol, and enhanced-bioavailability CoQ10 formulations were eligible when the independent effect of CoQ10 could be isolated.

PICOS component	Inclusion criteria	Exclusion criteria/boundary
Population	Adults in studies with a reported study-level mean or median age ≥40 years, or a separately extractable subgroup meeting that threshold. Community-dwelling, physically active, and clinical/disease-specific populations were eligible.	Mean/median age <40 years without a separately extractable eligible subgroup; pediatric populations.
Intervention	Oral CoQ10 as ubiquinone/ubidecarenone, ubiquinol, or an enhanced-bioavailability CoQ10 formulation; any dose; randomized exposure ≥2 weeks. Trials with balanced co-interventions were eligible. Multi-arm or factorial trials were eligible when an independent CoQ10 contrast could be isolated.	Non-oral administration; exposure <2 weeks; mitochondrial-targeted analogues or related compounds that are not CoQ10 (e.g., MitoQ/mitoquinone, idebenone, EPI-743); multi-ingredient products when the independent CoQ10 effect could not be isolated.
Comparator	Placebo, no supplementation, usual care, or the same background treatment/exercise program without CoQ10.	No concurrent eligible comparator, or unequal active co-interventions that prevented isolation of the CoQ10 effect.
Outcomes	At least one objective measure of: (1) muscle quantity/body composition (e.g., skeletal muscle mass, lean mass, fat-free mass, appendicular lean mass); (2) muscle strength, power, or endurance (e.g., handgrip, knee-extensor dynamometry, leg press); (3) physical performance or walking capacity (e.g., chair-stand, timed up-and-go, gait/walking tests, 6-minute walk distance); (4) exercise/cardiorespiratory capacity (e.g., VO_2peak/VO_2max, exercise duration, time to exhaustion, workload/power); or (5) a prespecified objective disease-specific motor-function scale. Trials known to have measured an eligible outcome but not reporting numerical results were retained for missing-evidence assessment.	Only biochemical/oxidative-stress markers, cardiac imaging, endothelial function, quality of life, fatigue, pain, or other self-reported symptoms without an eligible objective outcome.
Study design	Randomized controlled trials with parallel-group, crossover, or factorial designs. The randomized CoQ10 contrast had to be identifiable; crossover allocation had to preserve within-participant comparison.	Quasi-randomized or non-randomized studies; observational or single-arm studies; acute non-supplementation experiments; case reports; reviews; animal or in vitro studies; protocols or abstracts without outcome results.
Report characteristics	Full articles, theses, regulatory reports, or sufficiently detailed unpublished results. No language restriction. Multiple reports of one trial were linked and treated as one study.	Duplicate/secondary reports were not counted as independent studies. Reports with insufficient results were retained as awaiting information when appropriate but did not contribute quantitative data.

S2. Evidence routing

Eligibility for the systematic review was assessed before quantitative usability. Evidence class was assigned by outcome; therefore, one trial could contribute to different synthesis routes for different outcomes.

Class	Operational definition	Role
A - Core quantitative	Eligible effect estimate and variance directly reported or recoverable by standard algebra without an unverified correlation, digitization, or distributional conversion.	Primary meta-analysis
B - Assumption-dependent	Eligible, but quantitative inclusion requires a prespecified assumption or recovery method, such as an imputed paired correlation, graph digitization, median/range or IQR conversion, scale harmonization, or special handling of crossover carryover/washout.	Sensitivity meta-analysis only
C - Narrative	Eligible RCT, but an effect estimate/variance cannot be recovered with acceptable assumptions, or the reported objective outcome is too unique for a defensible pool.	Systematic review and narrative synthesis
D - Excluded	Fails one or more PICOS eligibility criteria.	Not included in the review

S3. Information sources and search dates

Five databases were searched from inception without language or publication-year restrictions. Search syntax and controlled vocabulary were adapted to each platform. Reference lists of eligible studies and relevant reviews were also screened.

Database	Date searched	Records	Known eligible reports retrieved	Limits
MEDLINE via PubMed	20 Jul 2026	1,190	32/33	No language/year limit; animal-only records excluded
Embase	21 Jul 2026	268	19/33	No language/year limit; animal-only records excluded
Web of Science Core Collection	21 Jul 2026	351	28/33	No language/year limit
Scopus	21 Jul 2026	541	29/33	No language/year limit
CENTRAL	21 Jul 2026	283	31/33	Trials collection
Combined exports	-	2,633	33/33	Count before cross-database deduplication

Known-item audit: after study selection, the 33 included reports were matched against the original database exports by DOI or normalized title. The combined exports recovered all 33 reports. Because the benchmark was derived from the completed review, this audit does not establish absolute sensitivity for unidentified or unindexed studies.

S4. Record management and study selection

Database records were exported with available bibliographic identifiers and deduplicated before screening. Two reviewers independently assessed titles and abstracts and then full texts against the prespecified criteria; uncertain records progressed to full-text review. Multiple reports from the same randomized trial were linked under one study identifier, and one principal reason for exclusion was recorded at full text. Quantitative usability was evaluated only after eligibility had been established; missing variance, crossover correlation, washout uncertainty, graph-only reporting, or statistical conversion affected the synthesis route rather than review eligibility.

S5. Exact electronic search strategies

The strategies below are reproduced as executed. Database-specific field tags and operators have been retained.

S5.1 MEDLINE via PubMed

Field	Details
Platform	PubMed
Date searched	20 July 2026
Coverage/limits	Database inception to the search date; no language or publication-year limit
Records retrieved	1,190 before deduplication

```
(
  "Ubiquinone"[Mesh:noexp]
  OR "coenzyme Q10"[tiab]
  OR "co-enzyme Q10"[tiab]
  OR "coenzyme Q-10"[tiab]
  OR "coenzyme Q 10"[tiab]
  OR coenzymeQ10[tiab]
  OR CoQ10[tiab]
  OR "Co-Q10"[tiab]
  OR ubidecarenone[tiab]
  OR ubiquinol[tiab]
)
AND
(
  randomized controlled trial[pt]
  OR controlled clinical trial[pt]
  OR randomized[tiab]
  OR randomised[tiab]
  OR randomly[tiab]
  OR placebo*[tiab]
  OR crossover[tiab]
  OR "cross-over"[tiab]
)
NOT (animals[mh] NOT humans[mh])
```

S5.2 Embase

Field	Details
Platform	Elsevier Embase.com Advanced Search
Date searched	21 July 2026
Coverage/limits	Database inception to the search date; no language or publication-year limit
Records retrieved	268 before deduplication

```
(
  'coenzyme q10'/exp
  OR
  (
    'coenzyme q10'
    OR 'coenzyme q-10'
    OR 'co-enzyme q10'
    OR 'co-enzyme q-10'
    OR coq10
    OR 'coq-10'
    OR ubiquinol
    OR 'ubiquinol-10'
    OR 'ubiquinone-10'
    OR 'ubiquinone 10'
    OR ubidecarenone
    OR 'reduced coenzyme q10'
    OR 'reduced coenzyme q-10'
  ):ti,ab,kw
)
AND
(
  'middle aged'/exp
  OR 'aged'/exp
  OR
  (
    (middle NEAR/1 age*)
    OR midlife
    OR 'mid-life'
    OR (older NEAR/1 adult*)
    OR (older NEAR/1 people)
    OR (older NEAR/1 person*)
    OR elderly
    OR aged
    OR aging
    OR ageing
    OR geriatric*
    OR 'late life'
  ):ti,ab,kw
)
AND
(
  'sarcopenia'/exp
  OR 'muscle mass'/exp
  OR 'muscle strength'/exp
  OR 'body composition'/exp
  OR 'exercise tolerance'/exp
  OR 'physical performance'/exp
  OR 'oxygen consumption'/exp
  OR 'motor performance'/exp
  OR
  (
    sarcopen*
    OR 'skeletal muscle mass'
    OR 'muscle mass'
    OR 'lean mass'
    OR 'lean body mass'
    OR 'fat free mass'
    OR 'fat-free mass'
    OR 'appendicular lean mass'
    OR 'appendicular skeletal muscle mass'
    OR 'muscle strength'
    OR 'muscular strength'
    OR handgrip
    OR 'hand grip'
    OR 'grip strength'
    OR 'muscle power'
    OR 'muscular power'
    OR torque
    OR 'physical function'
    OR 'physical functioning'
    OR 'physical performance'
    OR 'functional performance'
    OR 'functional capacity'
    OR 'exercise capacity'
    OR 'exercise tolerance'
    OR 'aerobic capacity'
    OR 'aerobic performance'
```

```
    OR 'cardiorespiratory fitness'
    OR vo2max
    OR 'vo2 max'
    OR vo2peak
    OR 'vo2 peak'
    OR 'peak oxygen uptake'
    OR 'maximal oxygen uptake'
    OR 'maximum oxygen uptake'
    OR 'oxygen consumption'
    OR 'six minute walk'
    OR '6 minute walk'
    OR 'six-minute walk'
    OR 6mwt
    OR 'walking distance'
    OR 'walking capacity'
    OR 'walking endurance'
    OR 'gait speed'
    OR 'walking speed'
    OR 'timed up and go'
    OR 'timed-up-and-go'
    OR tug
    OR 'chair stand'
    OR 'chair rise'
    OR 'sit to stand'
    OR 'sit-to-stand'
    OR 'short physical performance battery'
    OR sppb
    OR 'motor function'
    OR 'motor performance'
    OR 'motor examination'
    OR 'motor score'
    OR updrs
    OR 'unified parkinson disease rating scale'
    OR 'unified parkinsons disease rating scale'
    OR umsars
    OR 'unified multiple system atrophy rating scale'
    OR uhdrs
    OR 'unified huntington disease rating scale'
    OR psprs
    OR 'progressive supranuclear palsy rating scale'
  ):ti,ab,kw
)
AND
(
  'randomized controlled trial'/exp
  OR 'controlled clinical trial'/de
  OR 'randomization'/de
  OR 'double blind procedure'/de
  OR 'single blind procedure'/de
  OR 'triple blind procedure'/de
  OR 'crossover procedure'/de
  OR
  (
    random*
    OR placebo*
    OR trial
    OR crossover*
    OR 'cross over'
    OR ((single OR double OR triple OR treble) NEAR/1 blind*)
  ):ti,ab,kw
)
NOT
(
  'animal'/exp NOT 'human'/exp
)
```

S5.3 Web of Science Core Collection

Field	Details
Platform	Clarivate Web of Science, Advanced Search/Query Builder
Date searched	21 July 2026
Coverage/limits	Database inception to the search date; no language or publication-year limit
Records retrieved	351 before deduplication

```
#1 TS=("coenzyme Q10" OR "coenzyme Q 10" OR "co-enzyme Q10" OR CoQ10 OR "CoQ-10" OR ubiquinone OR "ubiquinone-10" OR
ubiquinol OR ubidecarenone OR "reduced coenzyme Q10")

#3 TS=(sarcopen* OR "skeletal muscle mass" OR "muscle mass" OR "lean mass" OR "lean body mass" OR "fat-free mass" OR
"fat free mass" OR "appendicular lean mass" OR "muscle strength" OR "muscular strength" OR handgrip OR "hand grip" OR
"grip strength" OR "muscle power" OR "muscle function" OR "muscular function" OR "physical performance" OR "physical
function" OR "physical functioning" OR "functional capacity" OR mobility OR "gait speed" OR "walking speed" OR "chair
stand*" OR "sit-to-stand" OR "sit to stand" OR "timed up and go" OR TUG OR "short physical performance battery" OR
```

```
SPPB OR "six-minute walk" OR "6-minute walk" OR 6MWT OR "exercise capacity" OR "exercise tolerance" OR "exercise
performance" OR "aerobic capacity" OR "cardiorespiratory fitness" OR "oxygen uptake" OR "oxygen consumption" OR
VO2max OR "VO2 max" OR VO2peak OR "VO2 peak" OR "exercise duration" OR "time to exhaustion" OR workload OR "work
capacity" OR "motor function" OR "motor performance" OR "motor symptom*" OR UPDRS OR "Unified Parkinson* Disease
Rating Scale" OR UMSARS OR "Unified Multiple System Atrophy Rating Scale" OR "ataxia rating" OR "disease-specific
motor")

#4 TS=(random* OR placebo* OR trial* OR crossover OR "cross-over" OR "cross over")

#5 #1 AND #3 AND #4
```

S5.4 Scopus

Field	Details
Platform	Elsevier Scopus Advanced Search
Date searched	21 July 2026
Coverage/limits	Database inception to the search date; no language or publication-year limit
Records retrieved	541 before deduplication

```
TITLE-ABS-KEY(
  "coenzyme Q10"
  OR "coenzyme Q 10"
  OR "co-enzyme Q10"
  OR "coenzyme-Q10"
  OR CoQ10
  OR "CoQ 10"
  OR "Co-Q10"
  OR ubidecarenone
  OR "reduced coenzyme Q10"
  OR "reduced coenzyme Q 10"
  OR "ubiquinone-10"
  OR "ubiquinone 10"
  OR "ubiquinol-10"
  OR "ubiquinol 10"
  OR "ubiquinol supplementation"
  OR "ubiquinol treatment"
  OR "oral ubiquinol"
  OR "ubiquinone supplementation"
  OR "ubiquinone treatment"
  OR "oral ubiquinone"
  OR "Q-ter"
  OR "Q-Gel"
  OR BPM31510
)
AND TITLE-ABS-KEY(
  randomized
  OR randomised
  OR randomly
  OR placebo
  OR "placebo controlled"
  OR "placebo-controlled"
  OR "double blind"
  OR "double-blind"
  OR "single blind"
  OR "single-blind"
  OR crossover
  OR "cross over"
  OR "cross-over"
)
AND TITLE-ABS-KEY(
  "muscle health"
  OR "muscle mass"
  OR "skeletal muscle mass"
  OR "lean body mass"
  OR "lean mass"
  OR "appendicular lean mass"
  OR "fat free mass"
  OR "fat-free mass"
  OR sarcopenia
  OR myopathy
  OR myalgia
  OR "muscle pain"
  OR "muscle symptom"
  OR "muscle symptoms"
  OR "muscle strength"
  OR "muscular strength"
  OR "muscle function"
  OR "muscle performance"
  OR "grip strength"
  OR handgrip
  OR "hand grip"
  OR "knee extension"
```

```
  OR "leg strength"
  OR "physical function"
  OR "physical functioning"
  OR "physical performance"
  OR "physical capacity"
  OR "functional capacity"
  OR "total functional capacity"
  OR "exercise capacity"
  OR "exercise performance"
  OR "exercise tolerance"
  OR "exercise duration"
  OR "exercise time"
  OR "aerobic capacity"
  OR "aerobic performance"
  OR endurance
  OR "work capacity"
  OR "peak work rate"
  OR "oxygen consumption"
  OR "oxygen uptake"
  OR "maximal oxygen uptake"
  OR "peak oxygen uptake"
  OR VO2max
  OR VO2peak
  OR "VO2 max"
  OR "VO2 peak"
  OR "walking distance"
  OR "walking speed"
  OR "gait speed"
  OR "six minute walk"
  OR "six-minute walk"
  OR "6 minute walk"
  OR "6-minute walk"
  OR "6MWT"
  OR "10 meter walk"
  OR "10 metre walk"
  OR "chair stand"
  OR "chair rise"
  OR "sit to stand"
  OR "sit-to-stand"
  OR "timed up and go"
  OR "short physical performance battery"
  OR SPPB
  OR "motor function"
  OR "motor performance"
  OR "motor score"
  OR UPDRS
  OR UHDRS
  OR UMSARS
  OR ICARS
  OR ataxia
  OR NYHA
)
```

S5.5 Cochrane Central Register of Controlled Trials (CENTRAL)

Field	Details
Platform	Cochrane Library Search Manager
Date searched	21 July 2026
Coverage/limits	Database inception to the search date; content type restricted to Trials
Records retrieved	283 before deduplication

Line	Search strategy
#1	MeSH descriptor: [Ubiquinone] explode all trees
#2	("coenzyme Q10" OR "coenzyme Q 10" OR "co-enzyme Q10" OR "co-enzyme Q 10" OR CoQ10 OR "CoQ-10" OR ubiquinone* OR ubiquinol* OR ubidecarenone OR "reduced coenzyme Q10" OR Qter OR "Q-ter" OR "Q-Gel"):ti,ab,kw
#3	#1 OR #2
#4	(sarcopen* OR myopeni* OR dynapeni* OR "skeletal muscle" OR "muscle mass" OR "lean mass" OR "lean body mass" OR "fat free mass" OR "fat-free mass" OR "appendicular lean mass"):ti,ab,kw
#5	((muscl* NEAR/3 (strength* OR power OR function* OR endurance OR performance OR weakness OR fatigue)) OR handgrip OR "hand grip" OR "grip strength" OR dynamomet* OR "knee extension" OR (knee NEXT extensor*) OR "leg press"):ti,ab,kw
#6	("physical performance" OR "physical capacity" OR "physical function" OR "functional capacity" OR "functional performance" OR "exercise capacity" OR "exercise tolerance" OR "exercise performance" OR "aerobic capacity" OR "aerobic fitness" OR "cardiorespiratory fitness" OR ((walk* OR gait OR mobility) NEAR/3 (test* OR distance OR speed OR time OR capacity OR endurance)) OR (six NEXT minute NEXT walk*) OR (6 NEXT minute NEXT walk*) OR 6MWT OR "timed up and go" OR "sit to stand" OR "sit-to-stand" OR "chair stand" OR VO2* OR "oxygen uptake" OR "oxygen consumption" OR (time NEAR/3 exhaustion) OR ((cycle* OR treadmill OR ergomet*) NEAR/3 (test* OR performance OR capacity OR endurance OR exhaustion))):ti,ab,kw

Line	Search strategy
#7	(UPDRS OR UHDRS OR ICARS OR SARA OR FARS OR "Unified Parkinson Disease Rating Scale" OR "Unified Huntington Disease Rating Scale" OR "International Cooperative Ataxia Rating Scale" OR "Friedreich Ataxia Rating Scale" OR ((Parkinson* OR Huntington* OR ataxia*) NEAR/5 (motor OR function* OR performance OR scale* OR score*))):ti,ab,kw
#8	#4 OR #5 OR #6 OR #7
#9	#3 AND #8
#10	#9 in Trials
