# Supplementary material for: Effects of Coenzyme Q10 Supplementation on Muscle Health and Physical Capacity in Middle-Aged and Older Adults: A Systematic Review and Meta-Analysis of Randomized Controlled Trials
Source: Antioxidants (Basel). 2026 Sep 22;15(10):1219. doi: 10.3390/antiox15101219 (PMC13700822; doi:10.3390/antiox15101219)
Supplement: Supplementary file 1 [file antioxidants-15-01219-s001.zip › 03_Supplementary_Figures_S1-S2_Evidence_Overview.pdf]

Figure S1. Included-evidence distribution map

Coverage across eight prespecified physical-capacity and muscle-health outcomes

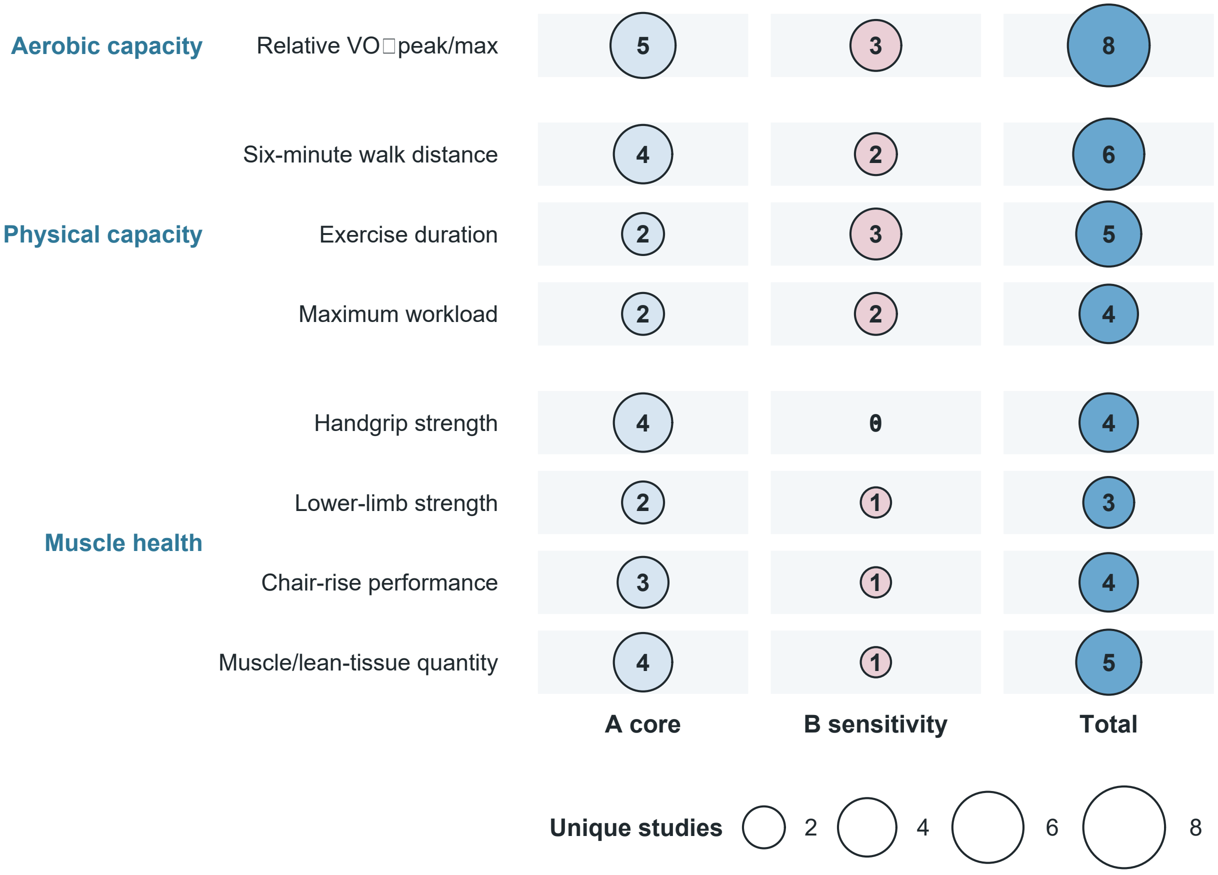

Numbers and bubble area indicate unique studies.
A, directly meta-analysable evidence; B, sensitivity/converted evidence.

Figure S2. Study-characteristics bubble plot

Each bubble represents one study contributing to at least one quantitative outcome

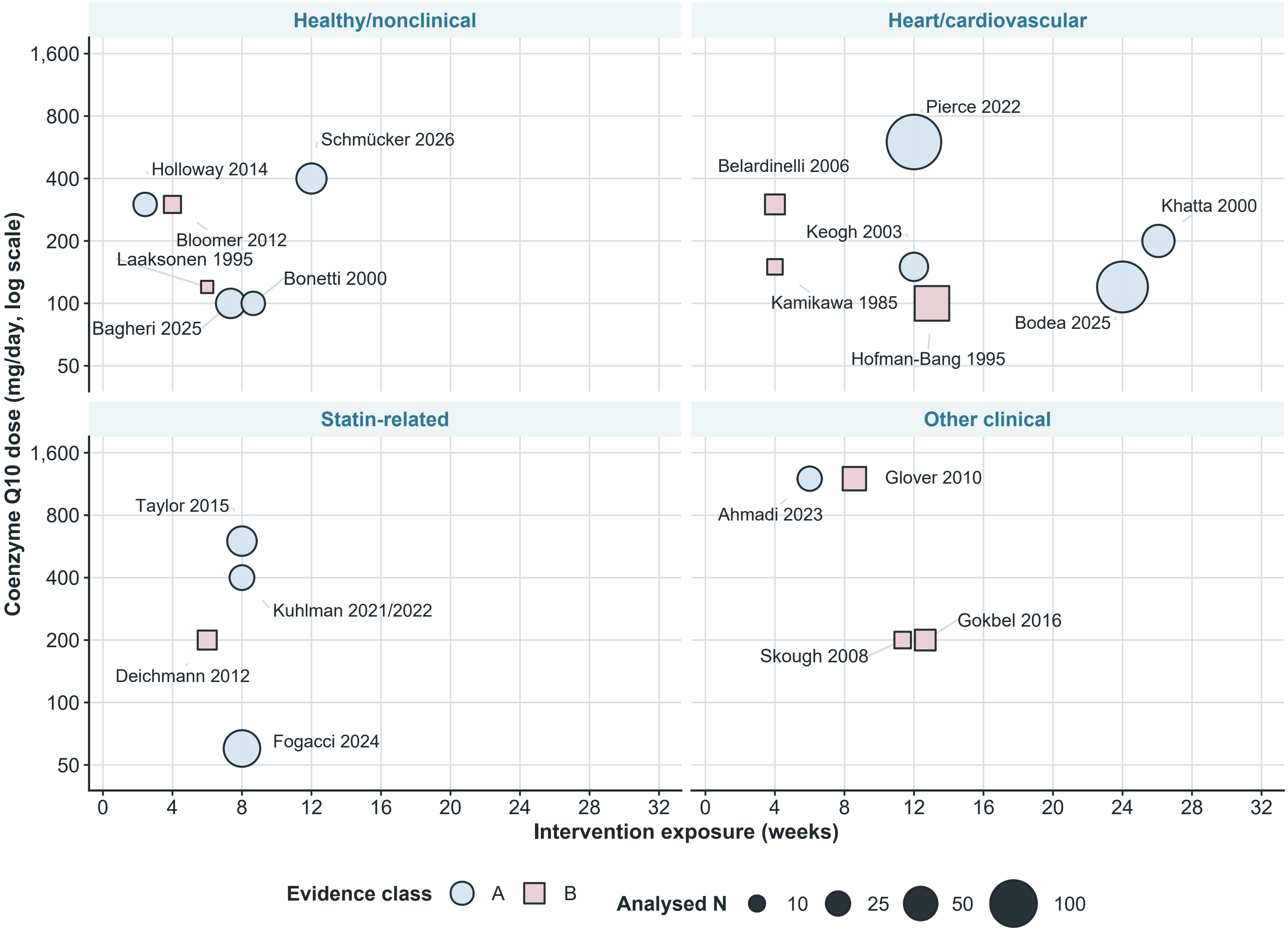

Bubble area represents analysed sample size; leader lines identify study labels.
Studies with identical coordinates are minimally offset for visibility. For crossover trials, duration is shown per intervention period.
