# Supplementary material for: Effects of Coenzyme Q10 Supplementation on Muscle Health and Physical Capacity in Middle-Aged and Older Adults: A Systematic Review and Meta-Analysis of Randomized Controlled Trials
Source: Antioxidants (Basel). 2026 Sep 22;15(10):1219. doi: 10.3390/antiox15101219 (PMC13700822; doi:10.3390/antiox15101219)
Supplement: Supplementary file 1 [file antioxidants-15-01219-s001.zip › 04_Supplementary_Figures_S3-S5_Study_Level_Forest_Plots.pdf]

Figure S3. Study-level traditional forest plot: Relative VO_2peak/max

Characteristics			Forest plot	Summary of findings	
**Study**	**Class**	**N**	**Random-effects model**	**Effect [95% CI]**	**Weight (%)**
**A primary analysis**					
Bonetti 2000	A	23		-3.00 [-9.51, 3.51]	1.5
Khatta 2000	A	46		0.70 [-1.00, 2.40]	22.3
Taylor 2015	A	38		1.00 [-3.20, 5.20]	3.7
Ahmadi 2023	A	25		0.67 [-0.33, 1.67]	65.0
Schmücker 2026	A	40		2.00 [-0.92, 4.92]	7.6
**Random-effects pooled**		172		**0.73 [-0.07, 1.54]**	**100.0**

Heterogeneity: I² = 0.0%, τ² = <0.01; overall p = 0.065; prediction interval [-0.07, 1.54]

Study	Class	N	Random-effects model	Effect [95% CI]	Weight (%)
**A + B sensitivity analysis**					
Bonetti 2000	A	23		-3.00 [-9.51, 3.51]	1.1
Khatta 2000	A	46		0.70 [-1.00, 2.40]	15.7
Taylor 2015	A	38		1.00 [-3.20, 5.20]	2.6
Ahmadi 2023	A	25		0.67 [-0.33, 1.67]	45.8
Schmücker 2026	A	40		2.00 [-0.92, 4.92]	5.3
Laaksonen 1995	B	7		-3.50 [-9.03, 2.03]	1.5
Belardinelli 2006	B	21		2.00 [0.04, 3.96]	11.8
Deichmann 2012	B	19		0.78 [-0.89, 2.45]	16.3
**Random-effects pooled**		219		**0.83 [0.09, 1.57]**	**100.0**

Heterogeneity: I² = 0.0%, τ² = <0.01; overall p = 0.033; prediction interval [0.09, 1.57]

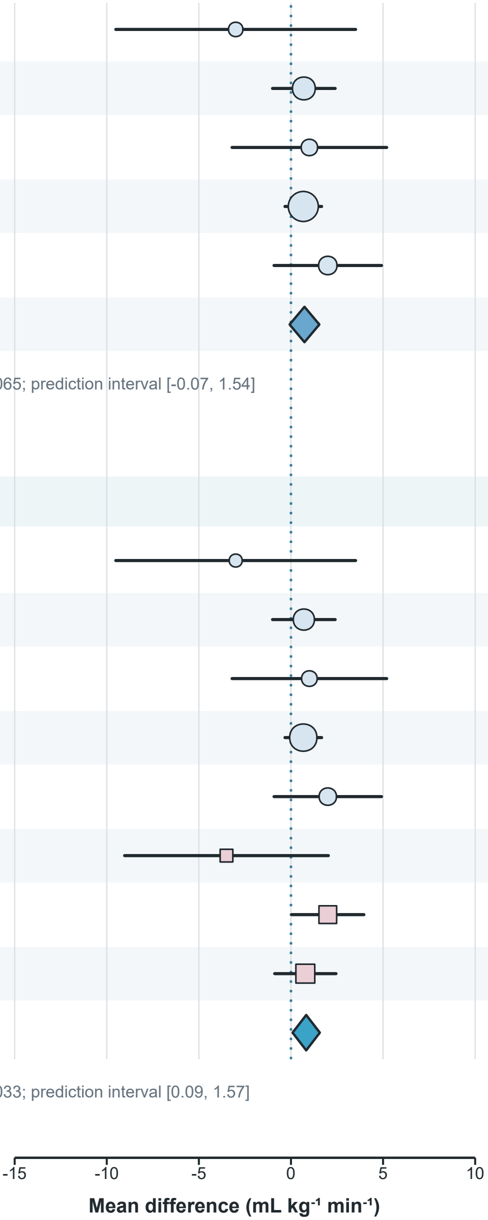

A, directly meta-analysable evidence; B, sensitivity/converted evidence. Circles denote A and squares denote B; point size reflects model weight. Diamonds represent Hartung–Knapp random-effects estimates.

Figure S4a. Study-level traditional forest plot: Six-minute walk distance

Characteristics			Forest plot	Summary of findings	
Study	Class	N	Random-effects model	Effect [95% CI]	Weight (%)
**A primary analysis**					
Keogh 2003	A	35		44.00 [-31.65, 119.65]	14.0
Pierce 2022	A	73		1.59 [-26.95, 30.13]	28.2
Bagheri 2025	A	38		20.95 [5.76, 36.14]	32.0
Bodea 2025	A	120		82.30 [47.03, 117.57]	25.9
**Random-effects pooled**		266		**34.58 [-22.56, 91.72]**	**100.0**

Heterogeneity: I² = 81.7%, τ² = 1046.4; overall p = 0.150; prediction interval not estimable

Study	Class	N	Random-effects model	Effect [95% CI]	Weight (%)
**A + B sensitivity analysis**					
Keogh 2003	A	35		44.00 [-31.65, 119.65]	9.9
Pierce 2022	A	73		1.59 [-26.95, 30.13]	21.0
Bagheri 2025	A	38		20.95 [5.76, 36.14]	24.3
Bodea 2025	A	120		82.30 [47.03, 117.57]	19.1
Skough 2008	B	14		-6.67 [-125.50, 112.17]	5.2
Gokbel 2016	B	23		-6.10 [-36.52, 24.32]	20.5
**Random-effects pooled**		303		**23.92 [-13.01, 60.85]**	**100.0**

Heterogeneity: I² = 77.3%, τ² = 919.3; overall p = 0.157; prediction interval [-62.33, 110.17]

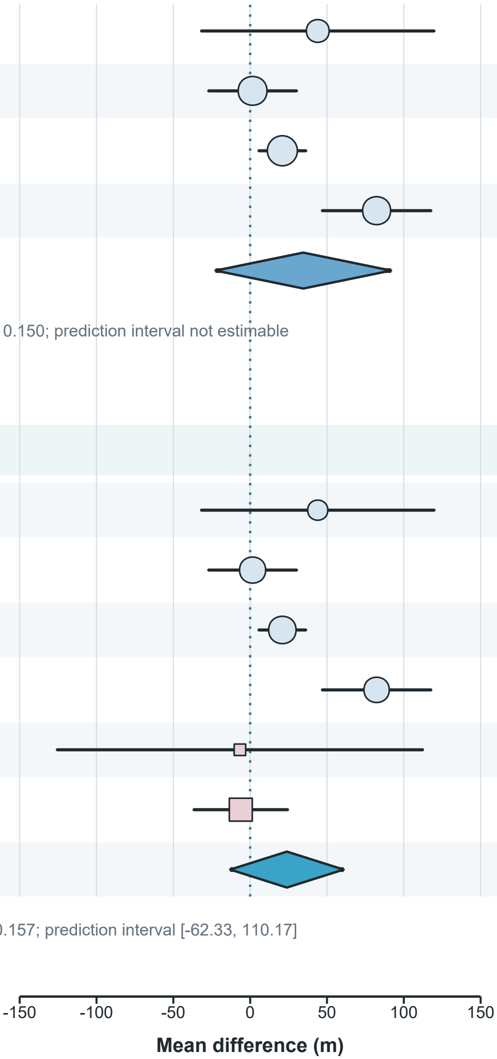

A, directly meta-analysable evidence; B, sensitivity/converted evidence. Circles denote A and squares denote B; point size reflects model weight.
Diamonds represent Hartung–Knapp random-effects estimates.

Figure S4b. Study-level traditional forest plot: Exercise duration

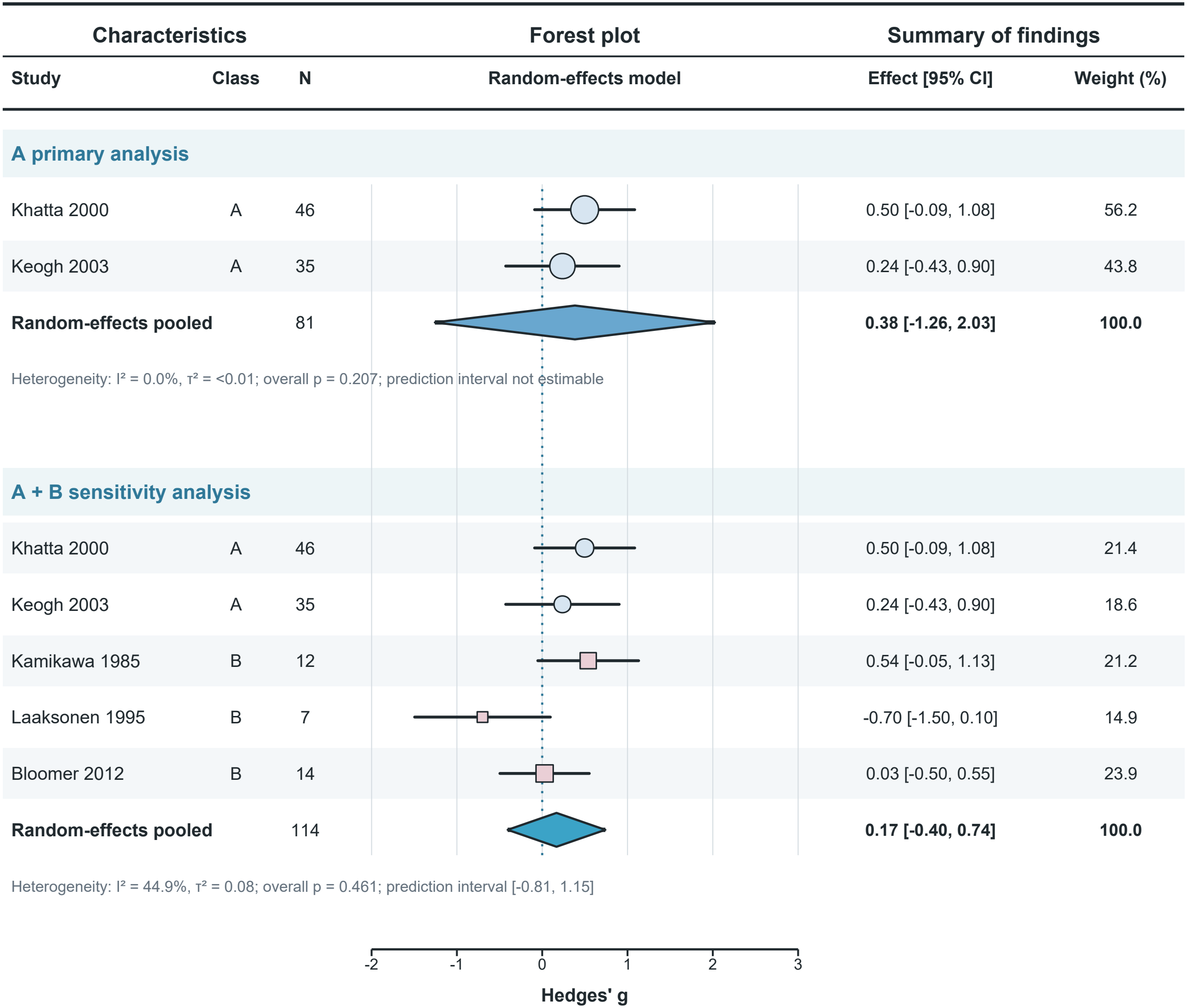

A, directly meta-analysable evidence; B, sensitivity/converted evidence. Circles denote A and squares denote B; point size reflects model weight. Diamonds represent Hartung–Knapp random-effects estimates. Class B recovery assumptions are reported in Supplementary Table S3.

Figure S4c. Study-level traditional forest plot: Maximum workload

Study	Class	N	Effect [95% CI]	Weight (%)
**A primary analysis**				
Bonetti 2000	A	23	31.00 [-12.41, 74.41]	38.8
Schmücker 2026	A	40	-6.00 [-32.49, 20.49]	61.2
**Random-effects pooled**		63	**8.34 [-220.71, 237.39]**	**100.0**
**A + B sensitivity analysis**				
Bonetti 2000	A	23	31.00 [-12.41, 74.41]	9.0
Schmücker 2026	A	40	-6.00 [-32.49, 20.49]	17.5
Hofman-Bang 1995	B	69	5.60 [1.15, 10.05]	38.3
Belardinelli 2006	B	21	25.00 [16.65, 33.35]	35.2
**Random-effects pooled**		153	**12.69 [-10.84, 36.22]**	**100.0**

A primary analysis: Heterogeneity: $I^2 = 50.8\%$, $\tau^2 = 348.0$; overall $p = 0.724$; prediction interval not estimable

A + B sensitivity analysis: Heterogeneity: $I^2 = 82.5\%$, $\tau^2 = 144.6$; overall $p = 0.185$; prediction interval not estimable

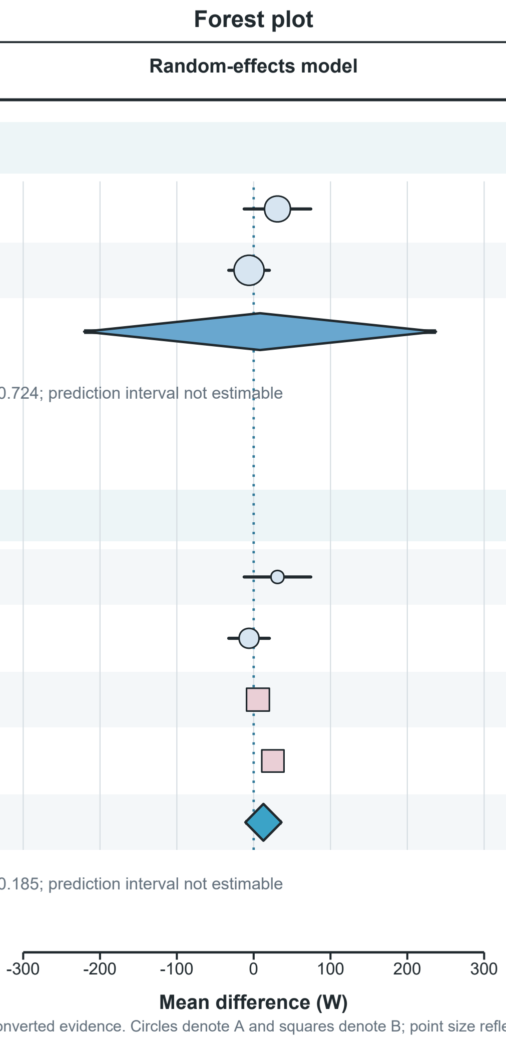

A, directly meta-analysable evidence; B, sensitivity/converted evidence. Circles denote A and squares denote B; point size reflects model weight.
Diamonds represent Hartung–Knapp random-effects estimates. Class B paired-correlation and washout decisions are reported in Supplementary Table S3.

Figure S5a. Study-level traditional forest plot: Handgrip strength

Characteristics			Forest plot	Summary of findings	
Study	Class	N	Random-effects model	Effect [95% CI]	Weight (%)
**A primary analysis**					
Taylor 2015	A	38		2.00 [-5.64, 9.64]	10.2
Kuhlman 2021/2022	A	19		0.10 [-4.05, 4.25]	20.6
Fogacci 2024	A	60		4.60 [4.32, 4.88]	36.0
Bagheri 2025	A	38		-0.07 [-1.47, 1.32]	33.3
**Random-effects pooled**		155		**1.85 [-2.08, 5.79]**	**100.0**

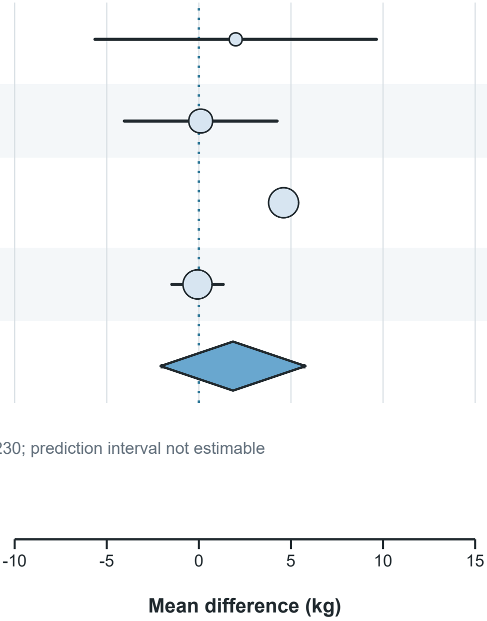

Heterogeneity: I^2 = 89.6%, τ^2 = 5.94; overall p = 0.230; prediction interval not estimable

A, directly meta-analysable evidence; B, sensitivity/converted evidence. Circles denote A and squares denote B; point size reflects model weight.
Diamonds represent Hartung–Knapp random-effects estimates.

Figure S5b. Study-level traditional forest plot: Lower-limb strength

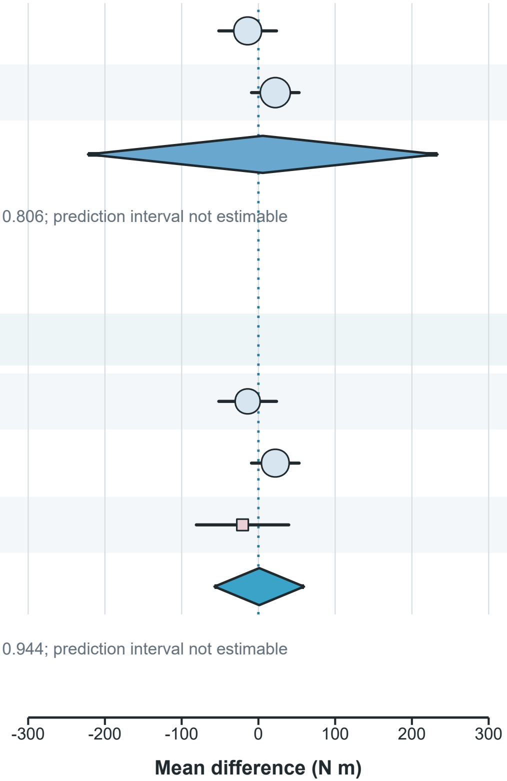

Study	Class	N	Effect [95% CI]	Weight (%)
**A primary analysis**				
Taylor 2015	A	38	-14.00 [-51.66, 23.66]	45.4
Kuhlman 2021/2022	A	19	22.00 [-9.06, 53.06]	54.6
**Random-effects pooled**		57	**5.64 [-222.12, 233.40]**	**100.0**
**A + B sensitivity analysis**				
Taylor 2015	A	38	-14.00 [-51.66, 23.66]	36.3
Kuhlman 2021/2022	A	19	22.00 [-9.06, 53.06]	45.3
Skough 2008	B	14	-20.67 [-80.91, 39.57]	18.4
**Random-effects pooled**		71	**1.07 [-57.28, 59.42]**	**100.0**

Heterogeneity (A primary analysis): $I^2 = 52.1\%$, $\tau^2 = 337.8$; overall $p = 0.806$; prediction interval not estimable

Heterogeneity (A + B sensitivity analysis): $I^2 = 34.2\%$, $\tau^2 = 225.3$; overall $p = 0.944$; prediction interval not estimable

A, directly meta-analysable evidence; B, sensitivity/converted evidence. Circles denote A and squares denote B; point size reflects model weight.
Diamonds represent Hartung–Knapp random-effects estimates.

Figure S5c. Study-level traditional forest plot: Chair-rise performance

Characteristics			Forest plot	Summary of findings	
Study	Class	N	Random-effects model	Effect [95% CI]	Weight (%)
**A primary analysis**					
Fogacci 2024	A	60		2.62 [1.93, 3.31]	33.2
Bagheri 2025	A	38		1.14 [0.46, 1.83]	33.2
Schmücker 2026	A	40		-0.28 [-0.90, 0.35]	33.6
**Random-effects pooled**		138		**1.16 [-2.45, 4.76]**	**100.0**
Heterogeneity: I² = 94.5%, τ² = 1.99; overall p = 0.301; prediction interval not estimable					
**A + B sensitivity analysis**					
Fogacci 2024	A	60		2.62 [1.93, 3.31]	25.4
Bagheri 2025	A	38		1.14 [0.46, 1.83]	25.4
Schmücker 2026	A	40		-0.28 [-0.90, 0.35]	25.7
Skough 2008	B	14		-0.53 [-1.60, 0.53]	23.5
**Random-effects pooled**		152		**0.76 [-1.55, 3.07]**	**100.0**
Heterogeneity: I² = 93.3%, τ² = 1.96; overall p = 0.373; prediction interval not estimable					

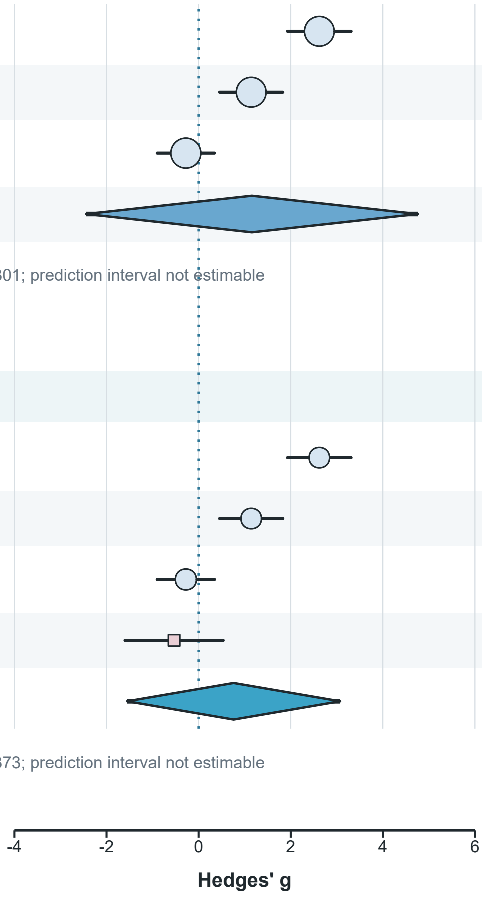

A, directly meta-analysable evidence; B, sensitivity/converted evidence. Circles denote A and squares denote B; point size reflects model weight.
Diamonds represent Hartung–Knapp random-effects estimates.

Figure S5d. Study-level traditional forest plot: Muscle/lean-tissue quantity

Characteristics			Forest plot	Summary of findings	
**Study**	**Class**	**N**	**Random-effects model**	**Effect [95% CI]**	**Weight (%)**
**A primary analysis**					
Holloway 2014	A	23		0.00 [-0.82, 0.82]	19.3
Kuhlman 2021/2022	A	19		0.44 [-0.47, 1.35]	15.5
Bagheri 2025	A	38		0.23 [-0.41, 0.86]	31.7
Schmücker 2026	A	40		-0.11 [-0.73, 0.51]	33.5
**Random-effects pooled**		120		**0.10 [-0.26, 0.47]**	**100.0**
Heterogeneity: I² = 0.0%, τ² = <0.01; overall p = 0.435; prediction interval not estimable					
**A + B sensitivity analysis**					
Holloway 2014	A	23		0.00 [-0.82, 0.82]	9.9
Kuhlman 2021/2022	A	19		0.44 [-0.47, 1.35]	8.0
Bagheri 2025	A	38		0.23 [-0.41, 0.86]	16.3
Schmücker 2026	A	40		-0.11 [-0.73, 0.51]	17.3
Glover 2010	B	28		0.01 [-0.36, 0.38]	48.5
**Random-effects pooled**		148		**0.06 [-0.15, 0.27]**	**100.0**
Heterogeneity: I² = 0.0%, τ² = <0.01; overall p = 0.472; prediction interval [-0.15, 0.27]					

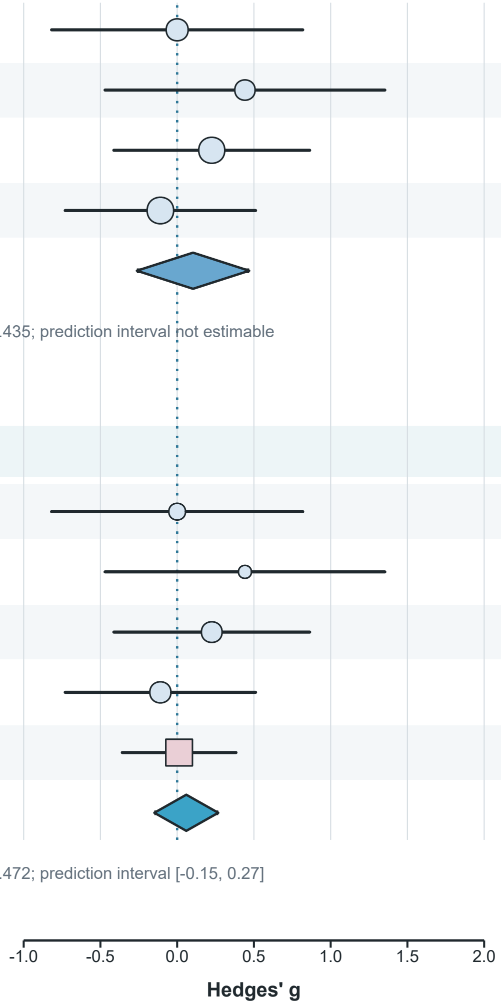

A, directly meta-analysable evidence; B, sensitivity/converted evidence. Circles denote A and squares denote B; point size reflects model weight.
Diamonds represent Hartung–Knapp random-effects estimates. Effects are reported as Hedges' g because skeletal muscle mass and lean mass are not directly exchangeable in kilograms.
