# Supplementary material for: Effects of Coenzyme Q10 Supplementation on Muscle Health and Physical Capacity in Middle-Aged and Older Adults: A Systematic Review and Meta-Analysis of Randomized Controlled Trials
Source: Antioxidants (Basel). 2026 Sep 22;15(10):1219. doi: 10.3390/antiox15101219 (PMC13700822; doi:10.3390/antiox15101219)
Supplement: Supplementary file 1 [file antioxidants-15-01219-s001.zip › 05_Supplementary_Figures_S6-S8_Sensitivity_and_Risk_of_Bias.pdf]

Figure S6a. Leave-one-study-out analysis: Relative VO_2peak/max

Circles represent the A-class model; squares represent the A+B augmented model. The full analysis is shown below the separator.

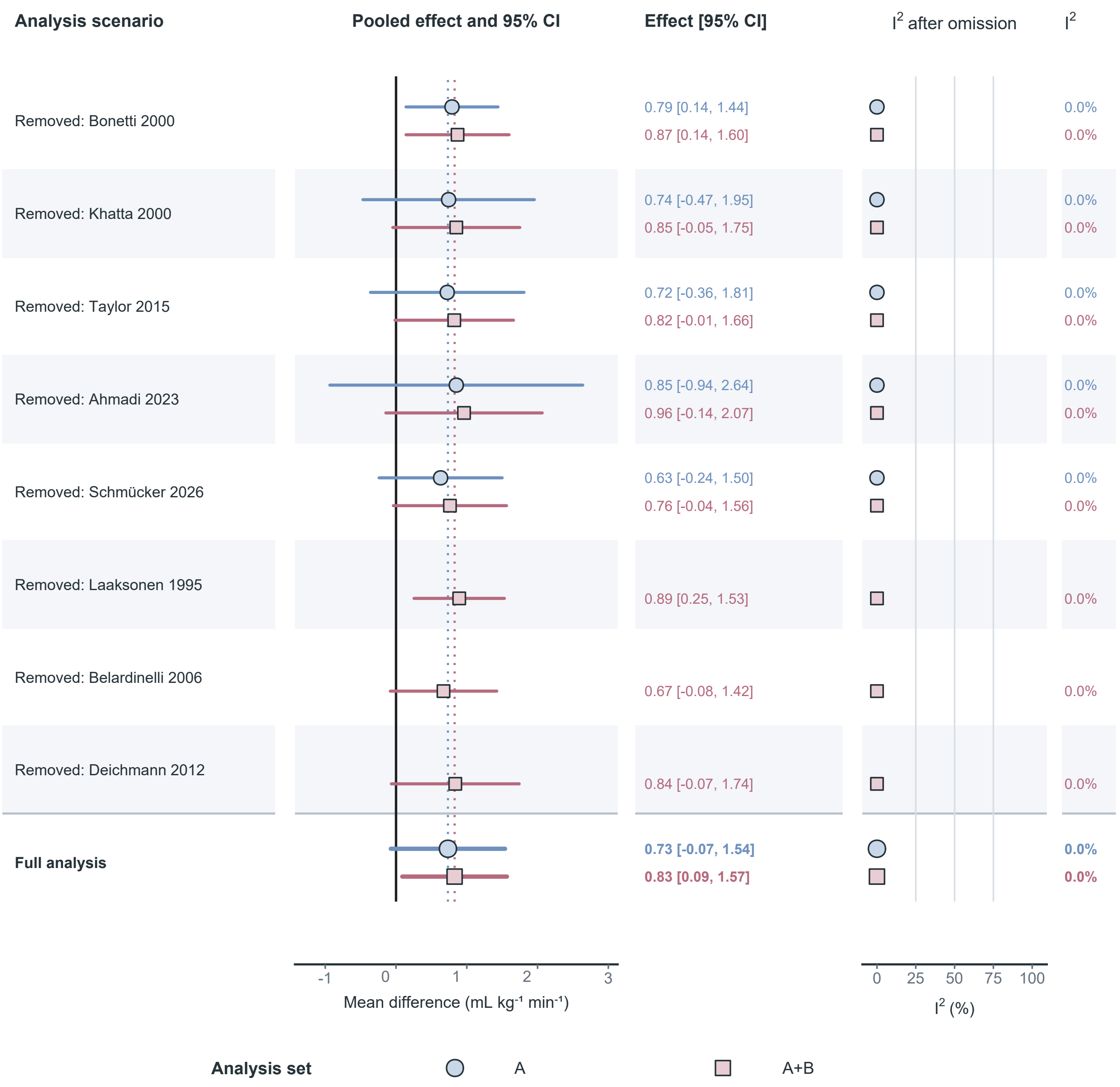

Figure S6b. Leave-one-study-out analysis: Six-minute walk distance

Circles represent the A-class model; squares represent the A+B augmented model. The full analysis is shown below the separator.

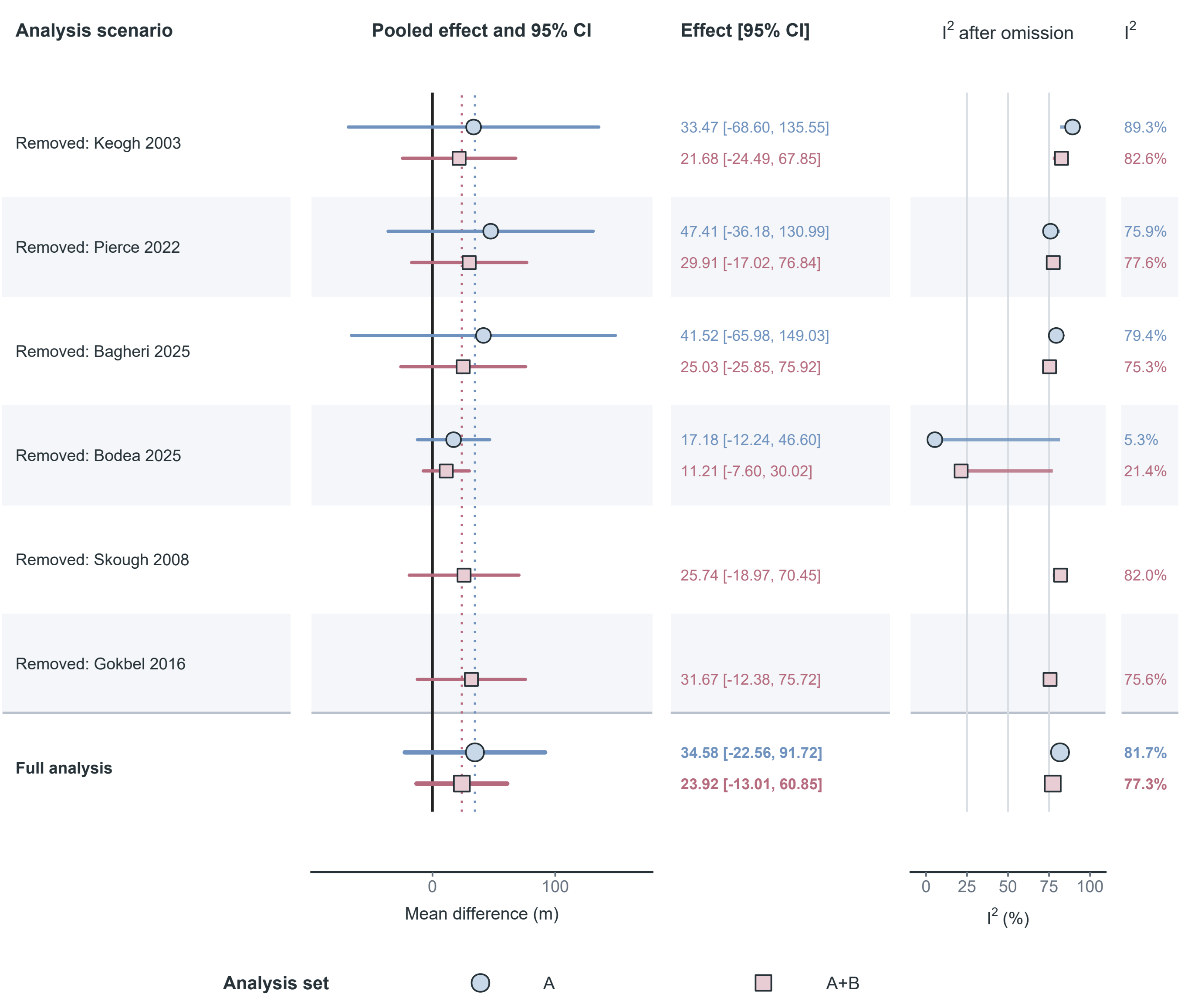

Figure S6c. Leave-one-study-out analysis: Exercise duration

Only the A+B model is shown because the A-only model contained fewer than three studies. The full analysis is below the separator.

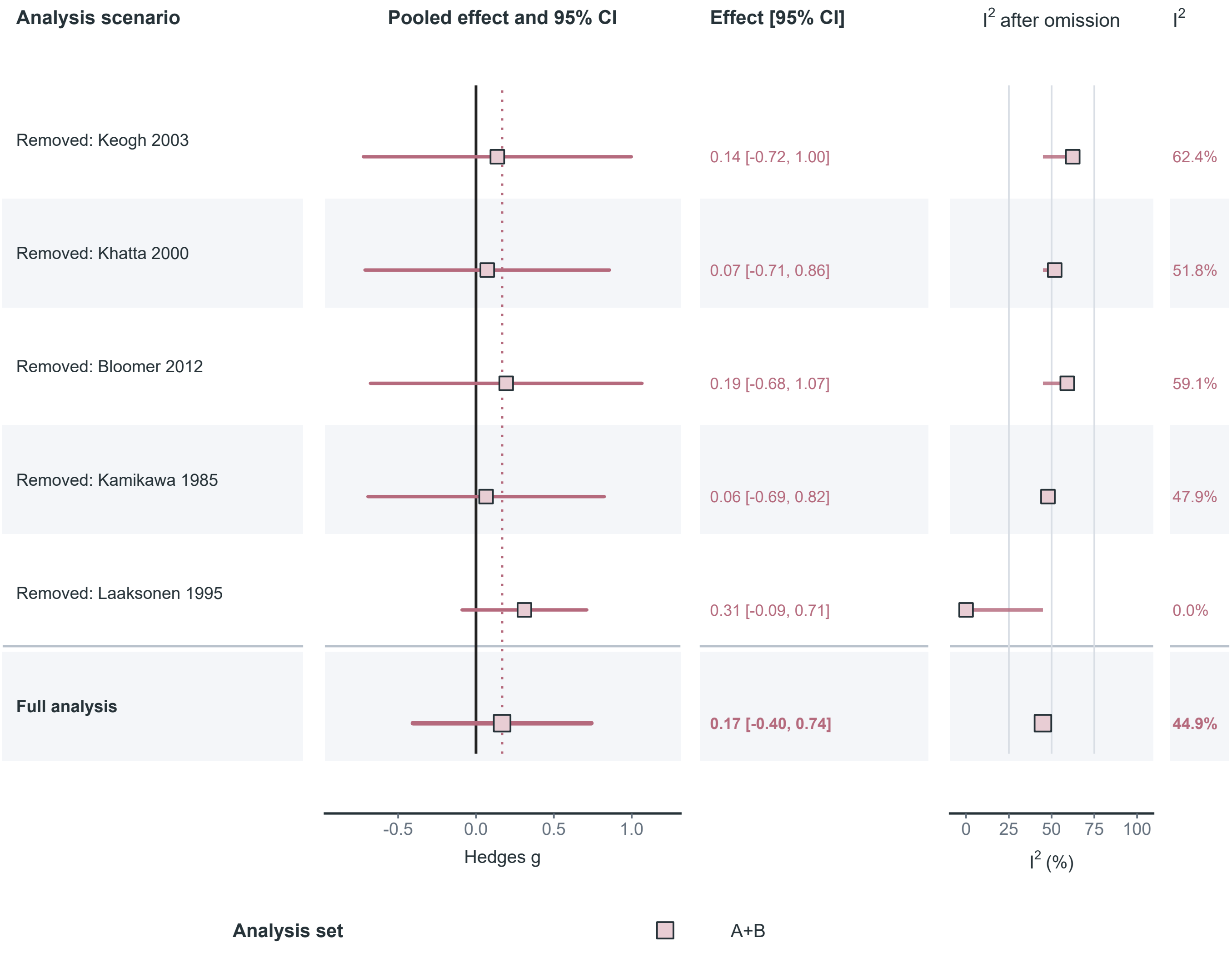

Figure S6d. Leave-one-study-out analysis: Maximum workload

Only the A+B model is shown because the A-only model contained fewer than three studies. The full analysis is below the separator.

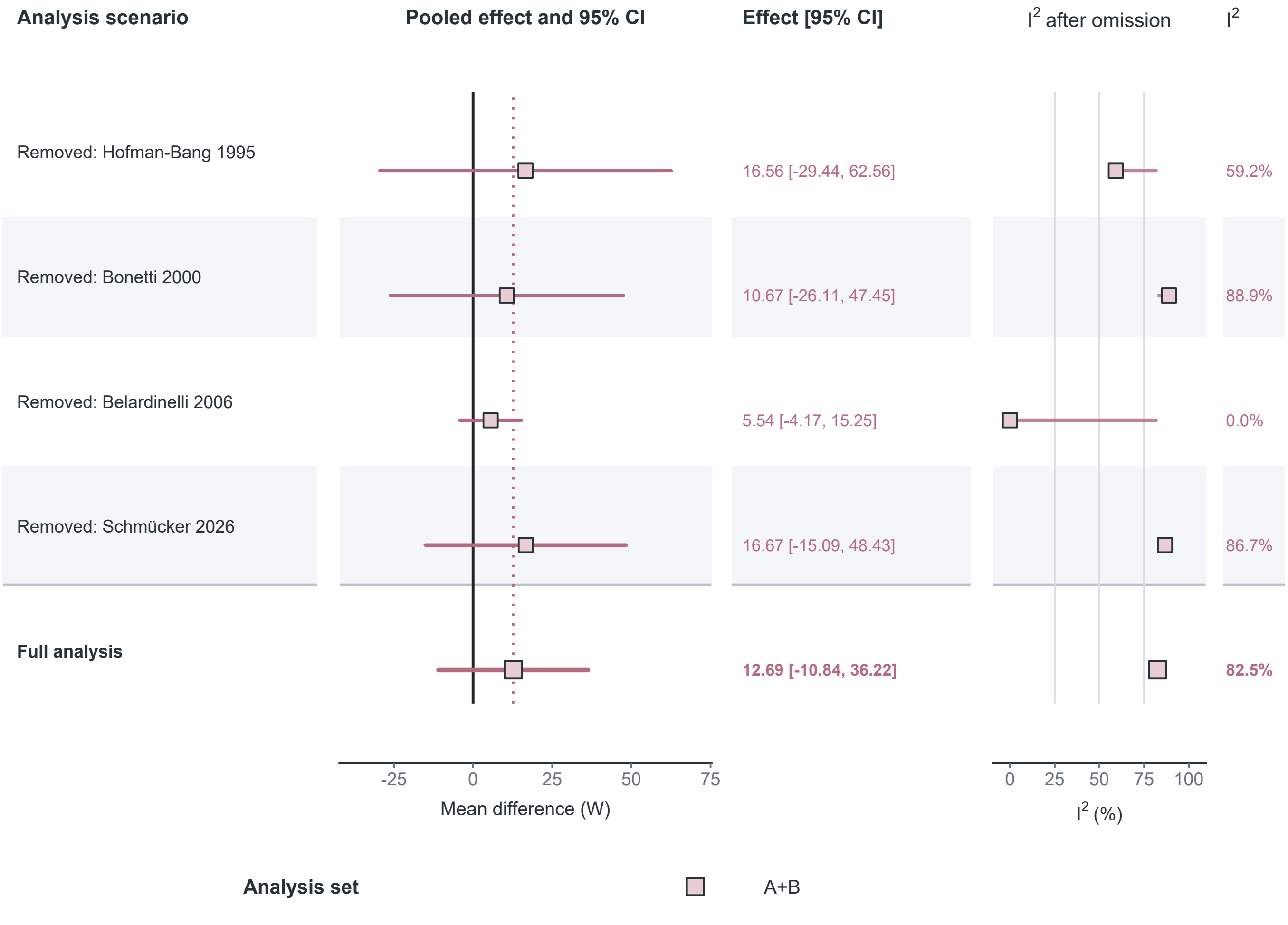

Figure S6e. Leave-one-study-out analysis: Handgrip strength

Only the A-class model is shown because no B-class study was available. The full analysis is below the separator.

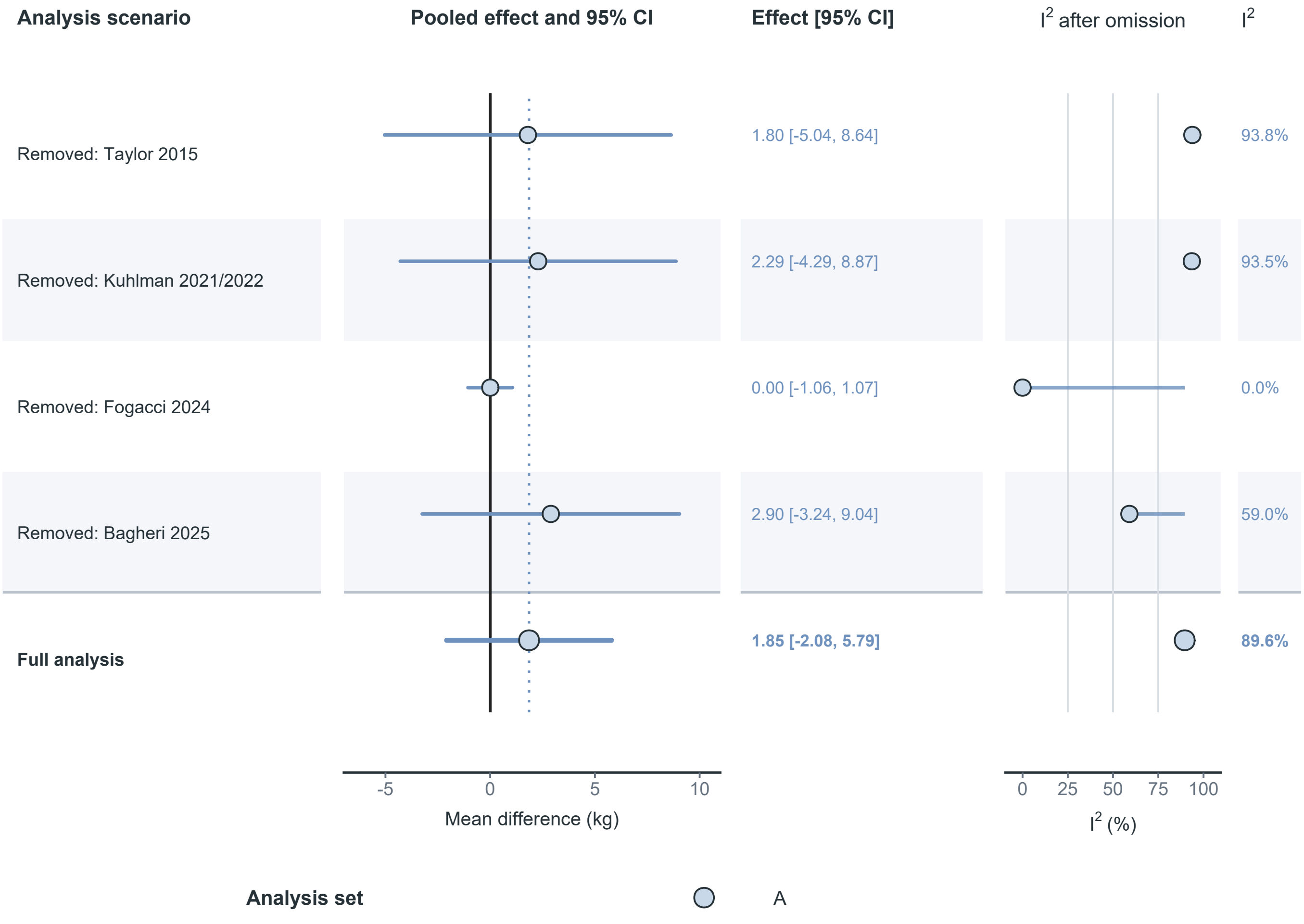

Figure S6f. Leave-one-study-out analysis: Lower-limb strength

Only the A+B model is shown because the A-only model contained fewer than three studies. The full analysis is below the separator.

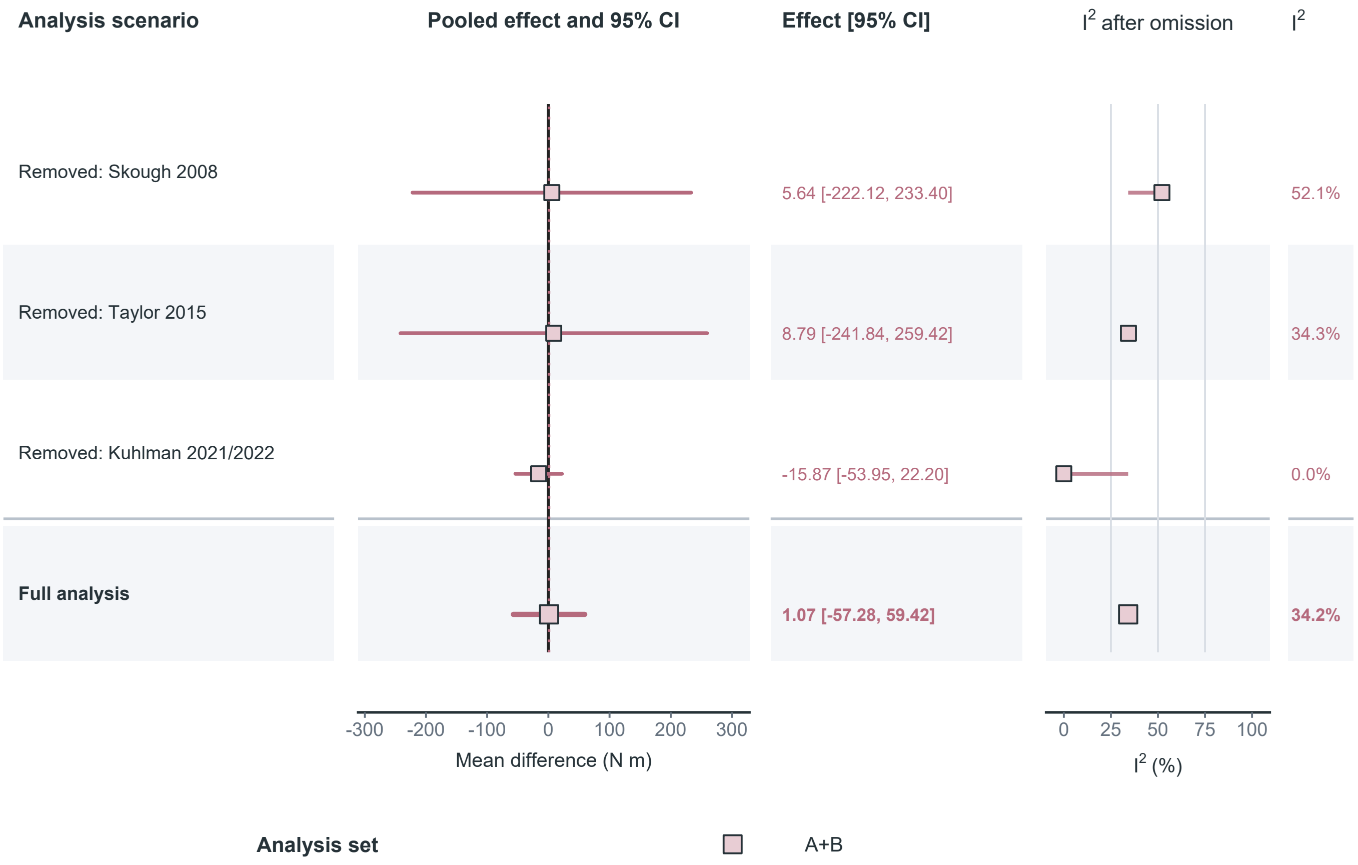

Figure S6g. Leave-one-study-out analysis: Chair-rise performance

Circles represent the A-class model; squares represent the A+B augmented model. The full analysis is shown below the separator.

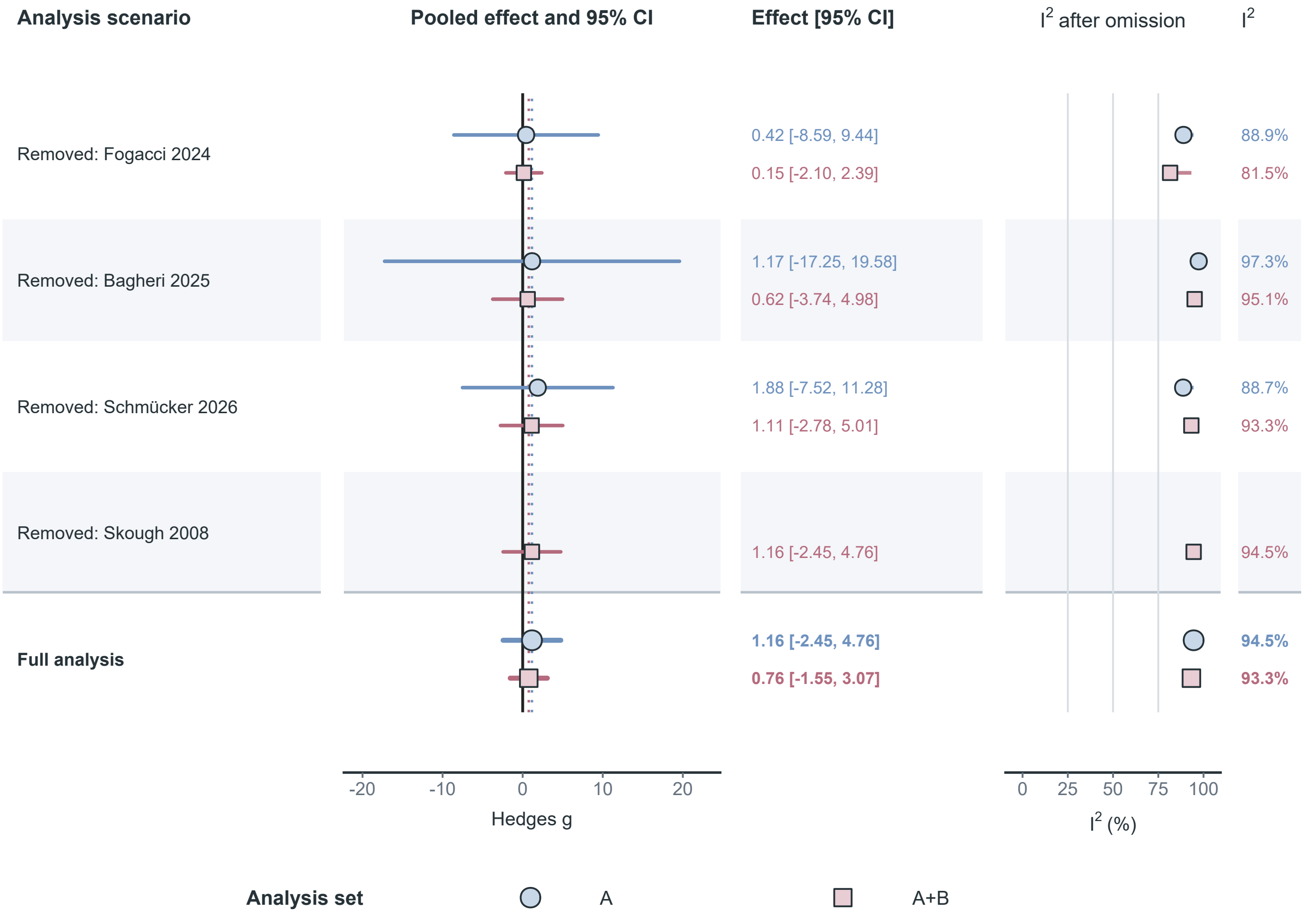

Figure S6h. Leave-one-study-out analysis: Muscle/lean-tissue quantity

Circles represent the A-class model; squares represent the A+B augmented model. The full analysis is shown below the separator.

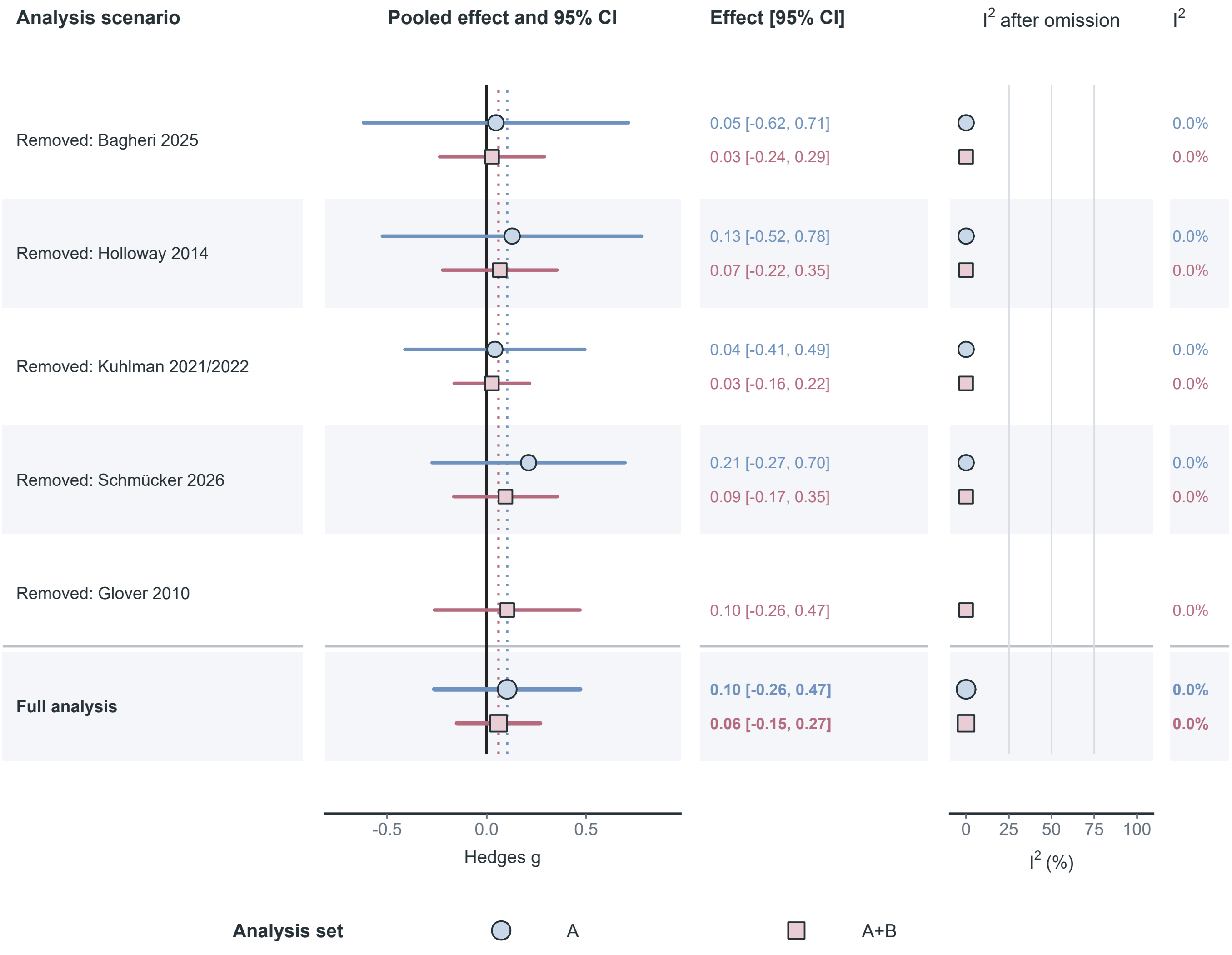

Figure S7. VO_2 heterogeneity stability after study removal

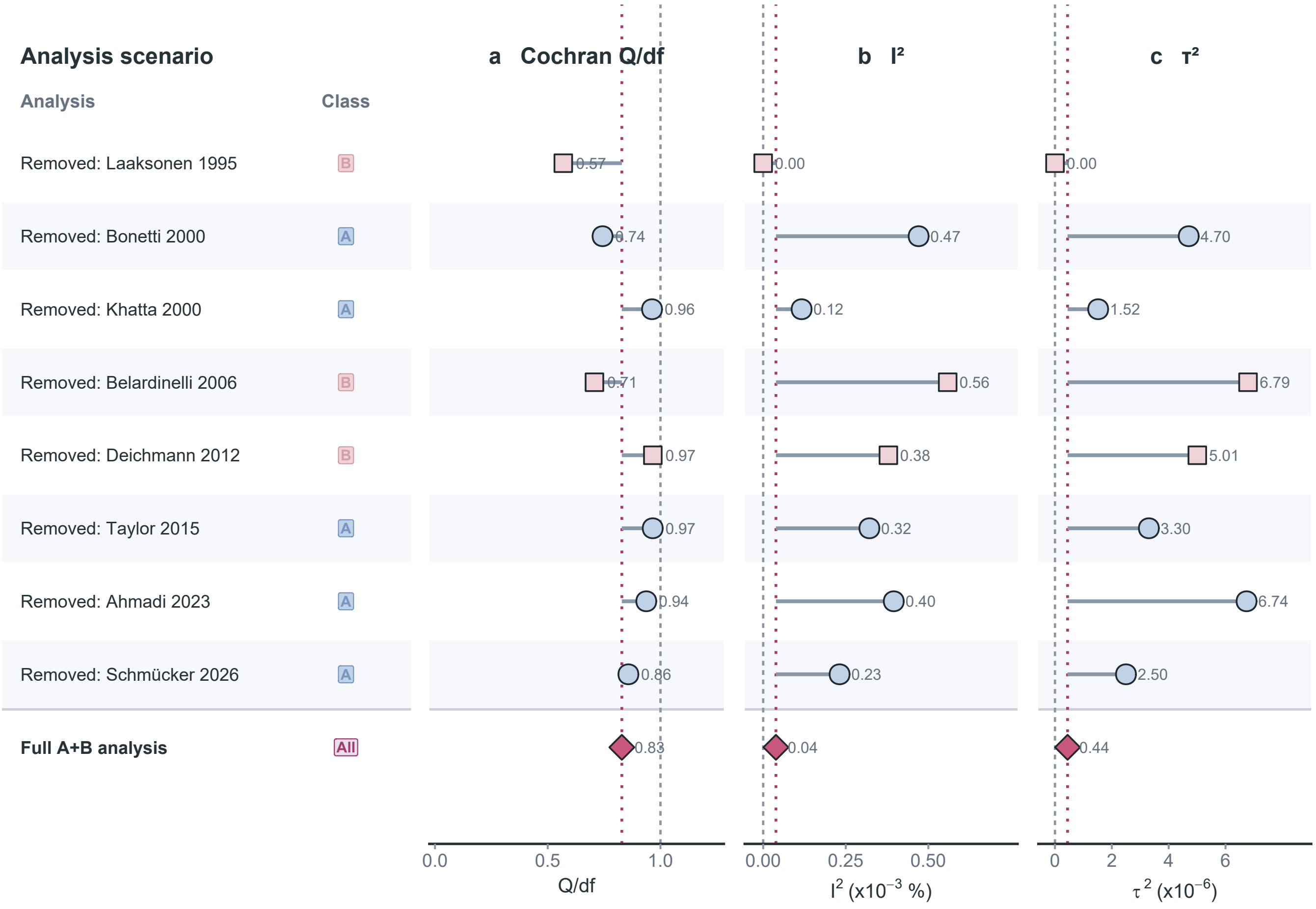

Figure S8. Study-level RoB 2 traffic-light plot

Judgments generated from the local Cochrane RoB 2 workbook; one principal eligible result per study

Study	Outcome assessed	D1	D2	D3	D4	D5	Overall
Ahmadi 2023	VO2peak / total work	?	+	+	+	+	?
Bagheri 2025	6MWD / handgrip / chair-rise	?	+	+	+	?	?
Beal 2014 (QE3)	UPDRS Part III motor score	?	+	+	+	+	?
Belardinelli 2006	VO2peak / peak workload	?	?	+	+	?	×
Berman 2004	6MWD	?	+	?	+	?	×
Bloomer 2012	Exercise duration	?	+	+	+	?	?
Bodea 2025	6MWD	?	+	+	+	×	×
Bonetti 2000	VO2peak / maximum workload	?	+	?	+	?	×
Deichmann 2012	Quadriceps repetitions	?	+	+	+	×	×
Fogacci 2024	Handgrip / 1-min sit-to-stand	+	+	+	+	+	+
Glover 2010	Handgrip / lean body mass	?	+	+	+	?	?
Gokbel 2016	6MWD / estimated VO2max	?	+	?	+	?	×
Golomb 2014	Lower-extremity Summary Performance Score	+	+	?	+	×	×
Hofman-Bang 1995	Maximum workload	?	?	?	+	?	×
Holloway 2014	Lean body mass	?	?	+	+	?	×
Huntington Study Group 2001 (CARE-HD)	UHDRS motor / functional capacity	+	+	+	+	?	?
Kamikawa 1985	Exercise duration	?	?	+	+	?	×
Keogh 2003	6MWD / Naughton exercise time	?	+	?	+	?	×
Khatta 2000	VO2peak / exercise duration	?	+	×	+	?	×
Kuhlman 2021/2022	VO2max / workload / strength / lean mass	?	+	?	+	+	?
Laaksonen 1995	VO2max / time to exhaustion in older subgroup	?	?	+	+	?	×
McGarry 2017 (2CARE)	UHDRS motor / functional capacity	?	+	?	+	+	?
Mitsui 2023	UMSARS Part II motor score / 10-m walk	+	+	+	+	+	+
Moghadam 2023	Walking time to exhaustion	?	+	+	+	+	?
Mortensen 2014 (Q-SYMBIO)	6MWD at 16 weeks	+	+	?	+	+	?
Müller 2003	UPDRS Part III motor score	?	+	+	+	?	?
Permanetter 1992	Maximum workload	?	?	+	+	×	×
Pierce 2022	6MWD (ubiquinol-only vs double-placebo)	+	+	?	+	+	?
Schmücker 2026	VO2max / workload / chair-rise / lean mass	?	+	+	+	?	?
Skough 2008	6MWD / chair-rise / knee-extensor performance	?	+	+	+	?	?
Stamelou 2008	PSP Rating Scale / UPDRS Part III	+	+	?	+	+	?
Taylor 2015	VO2max / handgrip / knee strength	+	+	+	+	?	?
Yoritaka 2015	UPDRS Part III motor score	?	+	?	+	?	×

+ Low ? Some concerns × High

D1: randomization process; D2: deviations from intended interventions; D3: missing outcome data; D4: outcome measurement; D5: selective reporting.
